# Inter and Intraspecific Genomic Divergence in *Drosophila montana* Shows Evidence for Cold Adaptation

**DOI:** 10.1093/gbe/evy147

**Published:** 2018-07-13

**Authors:** Darren J Parker, R Axel W Wiberg, Urmi Trivedi, Venera I Tyukmaeva, Karim Gharbi, Roger K Butlin, Anneli Hoikkala, Maaria Kankare, Michael G Ritchie

**Affiliations:** 1 Department of Biological and Environmental Science, University of Jyväskylä, Finland; 2 Center for Biological Diversity, School of Biology, University of St. Andrews, Fife, United Kingdom; 3 Department of Ecology and Evolution, University of Lausanne, Biophore, Switzerland; 4 Edinburgh Genomics, School of Biological Sciences, University of Edinburgh, United Kingdom; 5 Department of Animal and Plant Sciences, The University of Sheffield, UK; 6 Department of Marine Sciences, University of Gothenburg, Göteborg, Sweden; 7 Earlham Institute, Norwich Research Park, Norwich, United Kingdom

**Keywords:** cold tolerance, *Drosophila montana*, ecological adaptation, comparative genomics

## Abstract

The genomes of species that are ecological specialists will likely contain signatures of genomic adaptation to their niche. However, distinguishing genes related to ecological specialism from other sources of selection and more random changes is a challenge. Here, we describe the genome of *Drosophila montana*, which is the most extremely cold-adapted *Drosophila* species known. We use branch tests to identify genes showing accelerated divergence in contrasts between cold- and warm-adapted species and identify about 250 genes that show differences, possibly driven by a lower synonymous substitution rate in cold-adapted species. We also look for evidence of accelerated divergence between *D. montana* and *D. virilis*, a previously sequenced relative, but do not find strong evidence for divergent selection on coding sequence variation. Divergent genes are involved in a variety of functions, including cuticular and olfactory processes. Finally, we also resequenced three populations of *D. montana* from across its ecological and geographic range. Outlier loci were more likely to be found on the X chromosome and there was a greater than expected overlap between population outliers and those genes implicated in cold adaptation between *Drosophila* species, implying some continuity of selective process at these different evolutionary scales.

## Background

Comparative genomic analyses provide new insights into our understanding of evolutionary processes by helping to identify genes contributing to adaptive divergence (Ellegren 2008; Radwan and Babik 2012). If strong divergent selection due to environmental adaptation or social interactions, such as sexual selection, act as “barrier loci” by influencing species isolation, then identifying them can help to understand the process of speciation (Nosil et al. 2009; Smadja and Butlin 2011). However, accurately identifying such genes is a considerable challenge (Noor and Bennett 2009; Cruickshank and Hahn 2014; Ravinet et al. 2017; Wolf and Ellegren 2017).

Comparative genomic analyses are often hampered by a poor understanding of the sources of selection contributing to species divergence (Ravinet et al. 2017; Wolf and Ellegren 2017). Even when some of the sources of selection seem clear they are often complex and multifaceted, greatly complicating our ability to identify the genetic basis of adaptations. One approach to this problem is to apply comparative genomic techniques to species with distinct ecological specializations. Several studies have been made of such ecological specialists, including: cactophilic *Drosophila* (Matzkin et al. 2006; Smith et al. 2013), Asian longhorn beetles with specialized feeding habits (McKenna et al. 2016), climate-mediated adaptations in honey bees (Chen et al. 2016), and adaptation to high altitude in humans (Foll et al. 2014). These have successfully identified some associations, but such studies are still relatively few, hindering our general understanding of the genomic landscape of adaptation. Here, we describe the genome of *Drosophila montana*, a widely distributed northern member of the virilis group of *Drosophila*, which shows unique adaptations to seasonally varying environmental conditions prevailing at high latitudes and altitudes. *D. montana* is the most cold-tolerant *Drosophila* species known (Kellermann et al. 2012; Andersen et al. 2015). Their cold tolerance or hardiness involves multiple adaptations, including both a high general resistance to cold and a strong inducible cold acclimation response (Vesala and Hoikkala 2011), as well as a robust photoperiodic diapause (Tyukmaeva et al. 2011), which all contribute to its ability to survive through cold and dark winters. The daily and seasonal activity patterns of *D. montana*, and the interactions and neurochemistry of the core circadian clock genes behind these patterns, differs from those of more temperate species such as *D. melanogaster* (Kauranen et al. 2012, 2016; Tapanainen et al. 2018). These features have likely played an important role in allowing *D. montana* to colonize and persist in high-latitude environments (Terhzaz et al. 2015; Kauranen et al. 2016; Menegazzi et al. 2017).

*D* *rosophila* *montana* belongs to the *virilis* group of *Drosophila*, which comprises 13 species or subspecies divided into two clades, the virilis and montana phylads, the latter being further split into three lineages (Spicer and Bell 2002). These phylads are thought to have diverged in South Asia during the Early Miocene, after which both of them entered the New World by way of Beringia (Throckmorton 1982). The virilis phylad is constrained mostly within the temperate zone, whereas the montana phylad has expanded into a variety of habitats and spread to higher latitudes (Throckmorton 1982). Divergence of the two phylads has been estimated to have occurred 7 (Ostrega 1985) to 11 (Spicer and Bell 2002) Ma, whereas the North American, European, and Asian *D. montana* populations have diverged within the last 450,000–900,000 years (Mirol et al. 2007). Interestingly, conspecific *D. montana* populations have been shown to diverge in traits that play a role in ecological adaptation (e.g., Lankinen et al. 2013; Tyukmaeva et al. 2015), male sexual cues and female preferences (e.g., Klappert et al. 2007), and also to show sexual and postmating prezygotic reproductive barriers (Jennings et al. 2014). Information on potential candidate genomic regions and genes for traits involved in cold adaptation and sexual selection has been accumulated through QTL (Schäfer et al. 2010; Tyukmaeva et al. 2015), microarray (Vesala et al. 2012; Salminen et al. 2015), transcriptome (Parker et al. 2015; Kankare et al. 2016; Parker et al. 2016), and RNAi (Vigoder et al. 2016) studies.

Here, we aim to identify genes showing evidence of divergent selection linked to cold adaptation by contrasting the genomes of species and populations from different climatic conditions. These analyses were conducted at three levels. Firstly, we classified *Drosophila* species with well annotated genomes into cold-tolerant and non-cold-tolerant species and used branch tests to identify genes evolving differently between these contrasts. Secondly, we compared *D. montana* with its more temperate relative *D. virilis*. Finally, we compared three divergent populations of *D. montana* from different geographic regions. Such a multi-level approach allows us to identify genes that show recurrent divergence associated with climatic differences between species and populations. Such genes are likely to be particularly important for thermal adaptation, giving insight into the genes and functional processes involved in the evolution of cold tolerance in insects more generally. Thus, our results thus give a novel insight into genomic patterns of selection-driven divergence at different evolutionary scales, in addition to providing a well-annotated genome for a uniquely cold adapted insect species.

## Materials and Methods

### Samples and Sequencing

Genomic DNA for the *D. montana* reference genome was extracted from an inbred isofemale line originating from Vancouver, Canada (Can3F9) in summer 2003. This line was inbred via full-sib matings for 37 generations, relaxed for nine generations and maintained on malt food (Lakovaara 1969) at 19 °C in constant light. Quality checked DNA extracted from 210 males using a Gentra Puregene Tissue Kit (Qiagen) was used to produce three libraries with different insert sizes: 200, 400, and 3,000 bp. The 200 and 400 bp libraries were sequenced using an Illumina HiSeq 2000 at Edinburgh Genomics to produce paired-end reads (101 + 101 bp). The 3,000 bp library was sequenced using an Illumina MiSeq at The Centre for Genomic Research, University of Liverpool to produce mate-pair reads (101 + 101 bp). This strategy produced 65107854 paired-end reads for the 200 bp library, 25618163 paired-end reads for the 400 bp library and 19020110 mate-pair reads for the 3,000 bp library. Reads from the 200 and 400 bp libraries were trimmed using scythe (Buffalo 2014) to remove adaptors and sickle (Joshi and Fass 2011) to quality trim reads (bases with phred quality of <20 were trimmed from the tail end of each read). Reads from the 3,000 bp library were trimmed in the same manner, with the addition of a linker sequence removal step.

An initial assembly using reads from the 200 and 400 bp libraries was made using CLC assembly cell (4.0.12). Contigs from this were then blasted (blastN) to two subsets of NCBI’s nt database (arthropod and bacteria) with an *e*-value threshold of 1 × 10^−40^. Bit scores of blast hits from the arthropod and bacterial databases were compared for each contig, and any with a higher bit score for bacteria than arthropods were considered to be contaminants (supplementary fig. 9, Supplementary Material online). Reads were mapped to contigs identified as contaminants using BWA (v. 0.7.12) (Li and Durbin 2009) and then the unmapped reads were assembled using CLC assembly cell (4.0.12) (default options, minimum contig length = 200 bp). Contigs were then scaffolded using the 3,000 bp mate pair library using SSPACE-BASIC-2.0. This assembly contained 68,950 scaffolds (N50 = 39,341). This assembly was then further screened for contaminants using DeconSeq (v. 0.4.3) (Schmieder and Edwards 2011). Bacterial (2,786) and viral (4,359) genomes were downloaded from NCBI on January 20, 2016 and used as the contamination databases in DeconSeq along with the human genome (hg38). The *D. melanogaster* (r6.09) and *D. virilis* (r1.05) genomes were used as retention databases. DeconSeq identified 5,208 scaffolds as contaminants, which were removed from our assembly. We then used this assembly for all subsequent analyses. To assess the completeness of our genome assembly we used the CEGMA analysis pipeline (v. 2.4) (Parra et al. 2007, 2009) which identifies the presence of 248 conserved eukaryote genes, and the BUSCO pipeline (v.1.22) (Simão et al. 2015) which identifies the presence of 2,675 conserved arthropod genes.

### Genome Annotation

Full details of the genome annotation are given in the supplementary methods. Briefly, we used the Maker2 pipeline (Holt and Yandell 2011) to first mask putative repeats within the genome, and then used ab initio gene predictors SNAP and AUGUSTUS, and gene evidence (from protein homology and RNA-seq data) to generate gene predictions. Gene predictions from Maker2 were reciprocally blasted to proteins from *D. virilis* (r1.2) with the following cutoffs: *e*-value < 3 × 10^−13^, query cover >60% to give reciprocal best blast hits (RBBH). Orthologs for *D. melanogaster*, *D. sechellia*, *D. simulans*, *D. erecta*, *D. yakuba*, *D. ananassae*, *D. persimilis*, *D. pseudoobscura*, *D. willistoni*, *D. mojavensis*, and *D. grimshawi* were then obtained from FlyBase using *D. virilis* FlyBase numbers. Genes without a single ortholog for each species were discarded from multi-species selection analyses (below).

### Linkage Map Construction

For the genetic map construction, we selected 192 samples from a previous QTL study (Tyukmaeva et al. 2015), which consisted of two families (four parent individuals and their F2 progeny, females only). We used RAPiD Genomics’ (Florida, USA) facilities to develop a set of oligonucleotide probes for 13,975 selected regions in the largest scaffolds of the *D. montana* genome. These probes were used to capture sequence these target loci with 100 bp single end reads using an Illumina HiSeq 2000. A resulting SNP dataset was cleaned with Genotype Checker to eliminate possible errors in pedigree/genotyping (Paterson and Law 2011). The R/qtl package (Broman et al. 2003) was used to construct a genetic linkage map after discarding any polymorphic loci that were heterozygous for both parents, duplicated markers, markers showing segregation distortion, and individuals with fewer than 2,000 markers. Reads from the 200 and 400 bp genome reference libraries were mapped back to anchored scaffolds using BWA (v. 0.7.12) (Li and Durbin 2009). Multi-mapping reads were discarded. Since the genome reference libraries were produced from males, X linked regions should have half the coverage of autosomal regions, we used the coverage of these scaffolds to validate our linkage map.

### Selection Analyses

#### Multispecies Analysis

**Fig. 1.**
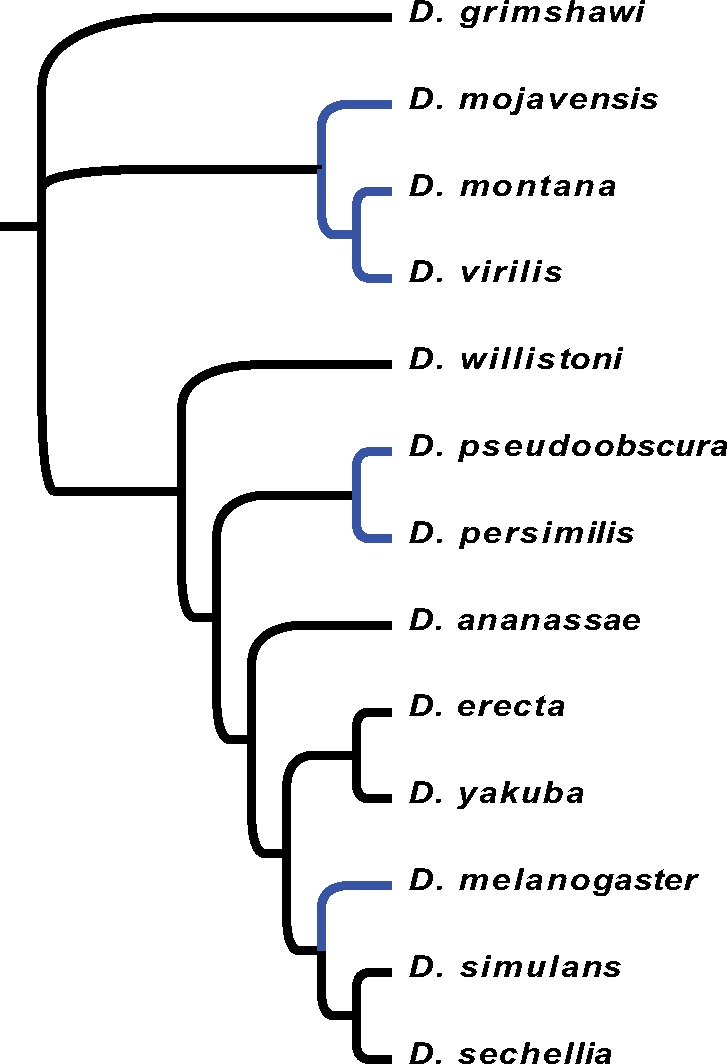
—Tree used for multi-species PAML analyses. Cold-tolerant species (species that have a knockdown temperature of <3 °C) are shown in blue (data from Kellermann et al. [2012] and MacMillan et al. [2015]).

13 species with fully annotated genome sequences available were divided into cold-tolerant and non-cold-tolerant ones; six species with a knockdown temperature <3 °C (Kellermann et al. 2012; MacMillan et al. 2015) were classified as cold-tolerant, the remainder as non-cold-tolerant fig. 1). This approach for classifying species was taken to a maximize the power of PAML’s branch tests (see below). To identify genes showing elevated signatures of selection in these species we extracted the longest CDS (*N* = 5,619) for each ortholog and codon-aligned them using PRANK (v.140110) (Löytynoja and Goldman 2005). Sequences were then analyzed in codeml from the PAML (v4.8) package (Yang 1997; Yang and Bielawski 2000). Two models were compared; the “null” model (clock = 0; fix_omega = 0, model = 0, NSSites = 0) which assumes a single common value for ω with an alternative model (clock = 0; fix_omega = 0, model = 2, NSSites = 0) which assumes one value of ω (dn/ds) for all the cold-tolerant species and a separate value of ω for the non-cold-tolerant species. Nested models were compared using a likelihood ratio test and *P* values corrected for multiple testing using a Bonferroni correction. Additionally, results were filtered to exclude sequences with dN, dS or ω > 10. This comparison tests whether there is a different rate of molecular evolution in cold-tolerant species compared with noncold-tolerant species.

#### Pairwise Analysis

To identify protein-coding genes with elevated signatures of selection we estimated pairwise ω (dN/dS) for each gene that had a reciprocal best blast hit (RBBH) to a *D. virilis* gene. The longest coding sequence of each gene and its RBBH ortholog were codon-aligned using PRANK (v.140110) (Löytynoja and Goldman 2005), before estimating ω using codeml in PAML (v. 4.8) (Yang 1997; Yang and Bielawski 2000). To determine if any genes showed ω > 1, we compared genes using a Bayesian estimation of ω in codeml (runmode = −3, model = 0, NSsites = 0) (Angelis et al. 2014) with default priors. The *P* values were corrected for multiple testing using a strict Bonferroni correction. We further filtered to exclude any genes where estimates of *dN, dS* or ω were >10.

We then compared mean ω values in several candidate gene sets (genes involved in immune function, reproduction, and cold tolerance) against the genomic background. Genes were classified into two “immune” classes firstly using the GO term “immune response” from FlyBase (version 6.05) and secondly using orthologs of genes identified as being involved in immune function by Sackton et al. (2007). Next, genes connected to reproduction were classified into several reproductive classes following Haerty et al. (2007): sex and reproduction related genes (SRR), female reproductive tract (FRTP) and seminal fluid proteins (SFP). Finally, cold tolerance genes were classified into two classes with genes differentially expressed in response to cold in *D. montana* and in *D. virilis* (Parker et al. 2015). Parker et al. (2015) found that from the differentially expressed genes, 42 were the same in both species but 550 were different, allowing genes to be classified into “cold tolerance same” and “cold tolerance different” groups.

#### Population Resequencing

For population comparisons we used *D. montana* flies from three populations: Oulanka (Finland; 66°N), Crested Butte, Colorado (USA; 39°N) and Vancouver (Canada; 49°N). These populations were established from the progenies of fertilized females collected in the summer of 2008 in Oulanka and Vancouver, and in the summer of 2009 in Colorado. Population cages were set up using 20 F3 generation individuals from approximately 20 isofemale lines for each population. Population cages were maintained at 19 °C in constant light (for more details see Jennings et al. [2011]). In March 2013, Genomic DNA was extracted from a pool of 50 females for each population and sequenced at Beijing Genomics Institute using an Illumina HiSeq 2000 to produce paired-end reads (90 + 90 bp, insert size = 500 bp).

Sequencing produced 84938118 paired-end reads for Colorado and 82663801 for Oulanka. Two runs for Vancouver resulted in 303365095 reads. Reads were quality trimmed (leading or trailing bases with a phred score of <20, or if two consecutive bases had an average phred score of <32 the read was trimmed at this point) and screened for adaptor sequence using trimmomatic (v. 0.30) (Bolger et al. 2014). Reads containing adaptor sequence or that had a length of <85 bp after quality trimming, were discarded. Since coverage depth can influence the estimation of allele frequency (Zhu et al. 2012), reads for Vancouver were randomly sampled prior to mapping to the mean number of reads from Colorado and Oulanka. Reads were mapped to the genome assembly using BWA (v. 0.7.12) (Li and Durbin 2009). Reads with a mapping quality of <20 were then removed, and an mpileup file was produced using samtools (v. 0.1.19) (Li et al. 2009). From this, a sync file was produced using PoPoolation2 pipeline (v 1.201) (Kofler et al. 2011). Outlier detection was performed on the raw read count data with BayeScan v. 2.1 (Foll and Gaggiotti 2008; Foll et al. 2010; Fischer et al. 2011), which performs comparably alongside other outlier methods in several simulation studies (Pérez-Figueroa et al. 2010; Vilas et al. 2012; Villemereuil et al. 2014). SNPs were filtered to include only sites with a minimum coverage of 25 and a maximum coverage of 93 (corresponding to the median 10th and 90th percentiles of the population coverage distributions). At the same time, SNPs were only considered if the minor allele had a read count >4 across all populations. BayeScan was run with five pilot runs of 1,000 iterations each followed by a main run of 2,000 iterations, a thinning interval of 10 and a burn in of 1,500. Additionally, three pairwise runs of BayeScan were performed with the same parameters as above. The three pairwise analyses compared Colorado to Vancouver, Vancouver to Oulanka, and Colorado to Oulanka populations, respectively.

### Functional Enrichment

To examine functional enrichment of genes for the species level selection analyses and population level *F*_ST_ scans, we used GOrilla (Eden et al. 2009). For the pairwise selection analyses genes were ranked by ω (from high to low and low to high). For the multispecies selection analyses, we ranked genes by *P* value and direction so that genes with the lowest *P* values and a higher ω in cold-tolerant species were at the top, and genes with lowest *P* values and higher ω in non-cold-tolerant species were at the bottom, allowing us to identify enriched GO terms for genes showing elevated ω in cold-tolerant species. To examine GO terms for genes showing elevated ω in non-cold-tolerant species the list order was simply reversed. For population level analyses genes were ranked by the most significantly differentiated SNP occurring within 1, 10, or 100 kb of a gene for each population. Results from GOrilla were then visualized using ReviGO (Supek et al. 2011), using the January 2017 version of Gene Ontology.

We used DAVID (v6.8) (Huang et al. 2009a, 2009b) to identify enriched functional groups of genes. A functional group was considered to be significantly enriched if its enrichment score (the geometric mean (in –log scale) of the *P* values of the GO terms in the group) was >1 (*P* < 0.1). For the pairwise selection analyses we identified functional clusters for genes occurring in the top and bottom 10% of genes for ω estimates. For the multispecies selection analyses we identified functional clusters for genes that showed a significantly (FDR < 0.1) higher ω in cold-tolerant species or in non-cold-tolerant species separately. For population level analyses we identified functional clusters for genes containing (within 1 kb) significantly differentiated SNPs for each population.

To take advantage of the superior annotation of *D. melanogaster* (Tweedie et al. 2009), we used *D. melanogaster* orthologs for all of the above function enrichment analyses. For the DAVID analyses the “background” list used was the subset of *D. melanogaster* genes available for each analysis.

## Results

### Genome Sequencing and Assembly

The assembled *D. montana* genome (table 1) has a total length of 183.6 Mb, which falls within the range seen for *Drosophila* species (111–187 Mb), and is similar to that of *D. virilis* (172 Mb), a close relative of *D. montana* with a sequenced genome. CEGMA identified 238 complete orthologs (96%) and 244 partial orthologs (98%) of the 248 CEGMA proteins and BUSCO identified 2,457 genes as complete (92%) and failed to identify only 46 (1.7%). RepeatMasker identified that 14.4% of the assembly was composed of repeat elements, the major classes of which were: Simple repeats (4.5%), LTR elements (4.3%), Unclassified (2.9%), and LINEs (1.9%) (supplementary fig. 1, Supplementary Material online). The total percentage of repeat elements identified was around half of that found for related *Drosophila* species (*D. virilis* = 25.9%, *D. mojavensis* = 23.8%, and *D. grimshawi* = 26.1%) likely reflecting the problem of assembling repetitive regions with short reads.

**Table 1:** Summary Statistics of *D. montana* Genome Assembly

Metric	Value
Total assembled length (bp)	183585048
Scaffolds (n)	63742
Scaffold N50 (bp)	40647
Largest scaffold (bp)	515352
GC content (%)	40.57
Number of predicted gene models	13683
Number of predicted gene models with RBBH to *D. virilis* genome	10898
CEGMA pipeline analysis (% complete/partial)	95.97/98.39
BUSCO (% complete/missing)	91.85/1.72

For the genetic map construction, the final dataset contained 5,858 polymorphic SNPs. The median depth of the SNPs in the final dataset was 52.4 and the average missing data rate was 0.003. The initial analysis formed five major linkage groups (as expected since *D. montana* has five chromosomes in total). Chromosome number was assigned by blasting genes assigned to the linkage groups to the *D. virilis* genome, which have been localized to chromosomes and is largely syntenic with *D. montana* (Schäfer et al. 2010). Although the analysis showed clear linkage groups, the order of markers was not totally resolved, likely due to lack of recombination events among F2 progeny (supplementary fig. 2, Supplementary Material online). The tentative scaffold order and position are given in supplementary table 1, Supplementary Material online. Using this map, we were able to anchor approximately one third of the genome assembly to chromosomes. To validate our linkage map, we examined coverage of anchored scaffolds. X-linked regions were found to have approximately half the coverage of autosomal regions, as expected since the reference genome was produced from male-only samples (supplementary fig. 3, Supplementary Material online).

### Between-Species Comparisons Identify Genes Showing Accelerated Divergence between Cold- and Warm Adapted Species

**Fig. 2.**
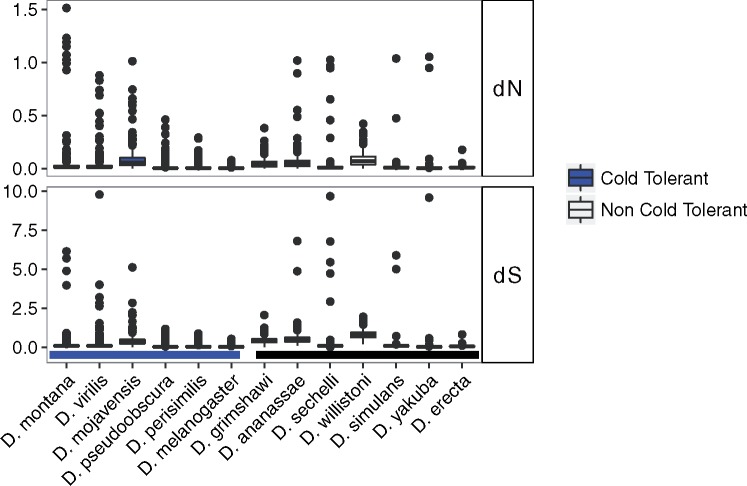
—Distributions of dN and dS estimates for each of the 250 genes from 13 *Drosophila* species with significant differences in ω between cold-tolerant and non-cold-tolerant species.

**Fig. 3.**
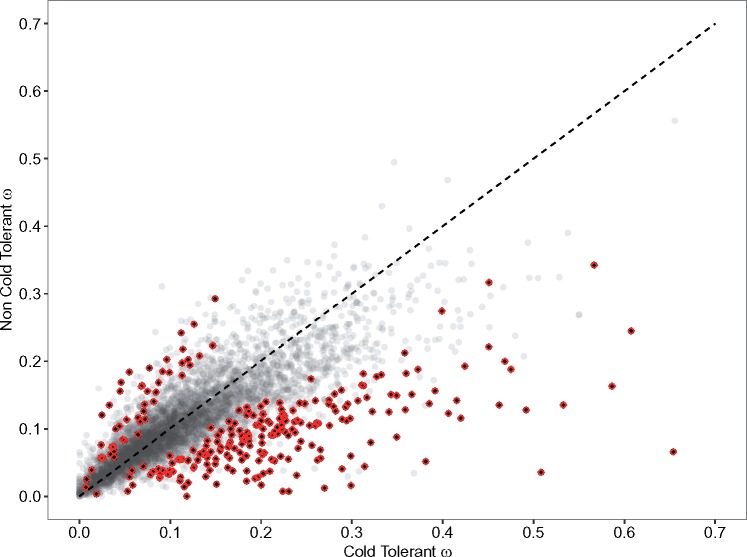
—The relationship between values of ω estimated for 5,619 genes in cold-tolerant and non-cold-tolerant species of *Drosophila*. 250 genes with significantly different estimates of ω are shown in black with red outline. Diagonal line indicates the 1–1 diagonal, points below the diagonal line show elevated levels of ω in cold-tolerant species compared with non-cold-tolerant species, whereas points above the diagonal show elevated levels of ω in non-cold-tolerant species.

**Fig. 4.**
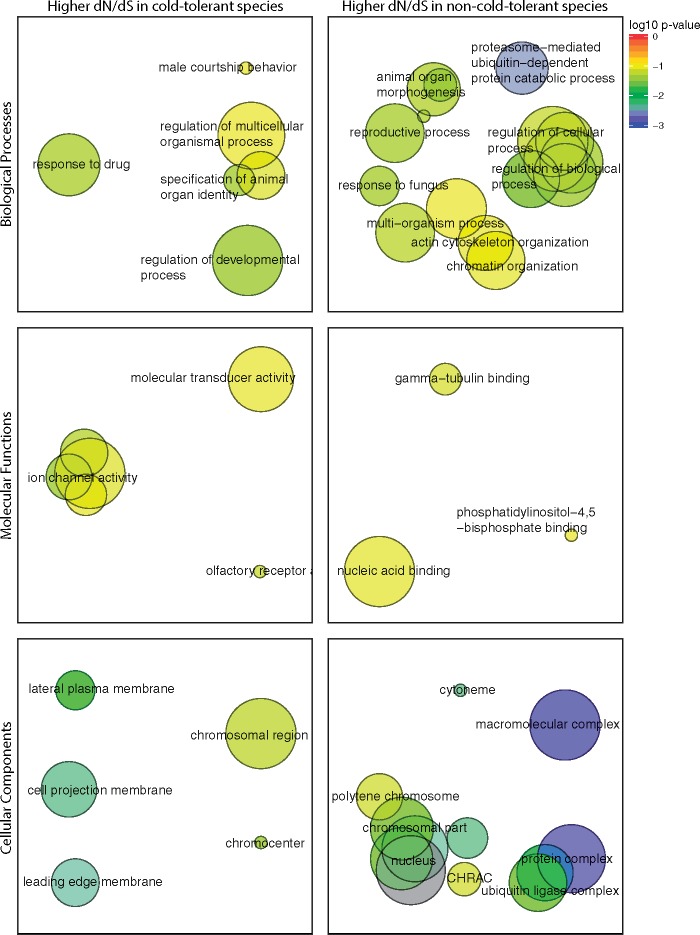
—Semantic clustering of significantly (FDR < 0.1) enriched GO-terms for genes showing significantly elevated dN/dS in cold-tolerant or non-cold-tolerant species. Circle size corresponds to the number of genes annotated to the term in the reference database. Circle colour indicates log_10_ FDR of the GO term.

Across the 13 *Drosophila* species we found 250 genes that had significantly different rates of evolution (ω) in cold- and non-cold-tolerant species (fig. 1 and supplementary table 2, Supplementary Material online). dS was on average lower for cold-tolerant species than for non-cold-tolerant species while dN was very similar (fig. 2 and supplementary table 3, Supplementary Material online). ω was on average greater for cold-tolerant species, probably driven by generally lower values of dS in these species (supplementary table 3, Supplementary Material online). 203 and 47 genes showed higher values of ω for cold-tolerant and for non-cold-tolerant species, respectively (fig. 3). Genes with elevated ω in cold-tolerant species were enriched for 23 GO terms (Biological Processes:Molecular Functions:Cellular Components = 6:10:7) (FDR < 0.1) (supplementary table 4, Supplementary Material online), which semantically cluster into the following categories: response to drug, male courtship behavior, olfaction, ion-channel activity, and developmental processes (fig. 4). Of genes with elevated ω in non-cold-tolerant species, we identified 50 enriched GO terms (Biological Processes:Molecular Functions:Cellular Components = 34:3:13) (FDR < 0.1) (supplementary table 5, Supplementary Material online), which semantically cluster into the following categories: proteasome-mediated ubiquitin-dependent protein catabolic process, reproductive processes, response to fungus, animal organ morphogenesis, regulation of biological, and cellular processes (fig. 4). Moreover, DAVID identified 11 functional group clusters for genes with significantly higher ω in cold-tolerant species (supplementary table 6, Supplementary Material online) including: Nucleotide-binding, Olfaction, Transmembrane proteins, Neural development, Leucine-rich repeat containing proteins, GTPase/GTP binding, Cytoskeleton/Microtubule, and Ion Transport. Finally, DAVID identified three functional group clusters for genes with significantly higher ω in cold-tolerant species (supplementary table 7, Supplementary Material online) including: Calcium-binding EGF domain containing proteins, Transmembrane proteins, and Cytoskeleton/Microtubule.

### Comparison of *D. montana* and *D. virilis*

**Fig. 5.**
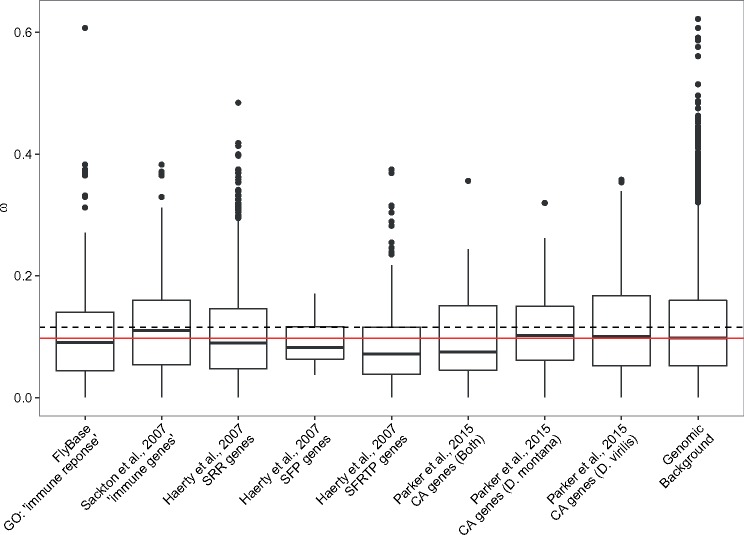
—Average values of ω between *D. montana* and *D. virilis* for candidate gene sets. FRTP = female reproductive tract SFP = seminal fluid proteins SRR = sex and reproduction related genes CA = cold acclimation genes. The red and dashed lines indicate the median and mean ω of the genomic background, respectively.

**Fig. 6.**
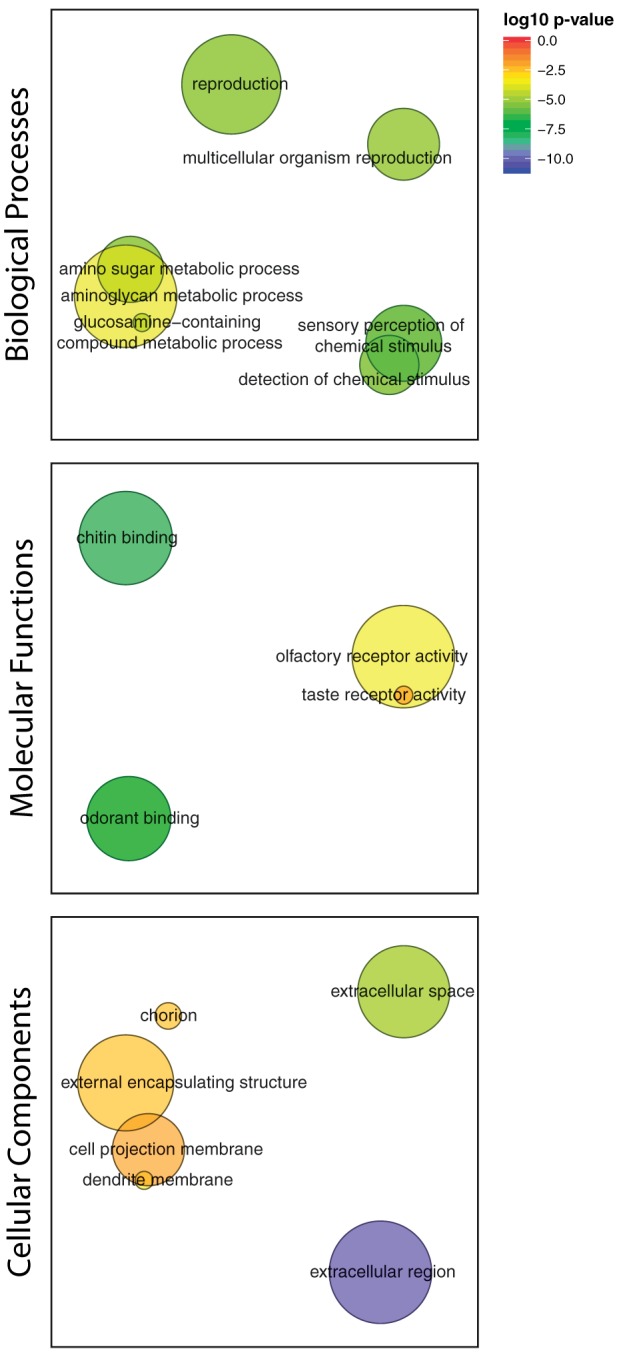
—Semantic clustering of significantly (FDR < 0.1) enriched GO-terms for genes showing high dN/dS between *D. montana* and *D. virilis*. Circle size corresponds to the number of genes annotated to the term in the reference database. Circle colour indicates log_10_ FDR of the GO term.

We estimated ω (dN/dS) for each of the one-to-one orthologs between *D. montana* and *D. virilis* (supplementary table 2, Supplementary Material online). No genes had a ω significantly >1 after filtering and multiple-test correction. Comparison of mean ω for several candidate gene sets (genes involved in immune function, reproduction, and cold tolerance) found that none of the candidate genes sets differed significantly from the genomic background (fig. 5). By ranking genes by ω we identified GO terms enriched in genes with relatively high and low ω. For those with high ω we identified 23 enriched GO terms (Biological Processes:Molecular Functions:Cellular Components = 10:4:9) (FDR < 0.1) (supplementary table 8, Supplementary Material online). Semantic clustering of these GO terms shows that they fall into the following categories: Reproduction, detection of chemical binding/olfaction, amino sugar metabolism, and chitin binding (fig. 6). DAVID identified nine functional group clusters (supplementary table 9, Supplementary Material online) including two related to chitin production and two related to olfactory functions, congruent with the findings from the single GO term enrichment analysis (above). In addition, DAVID also identified two clusters involved in: immune defense (C-type lectin domain carrying genes, and Fibrinogen related genes), Transcription factor binding, and a cluster containing genes with either a CAP (cysteine-rich secretory protein) or SCP (Sperm-coating protein) domain. We identified 662 enriched GO terms for genes with low ω between *D. montana* and *D. virilis* (Biological Processes:Molecular Functions:Cellular Components = 485:80:97) (FDR < 0.1). As expected for genes with very low ω the enriched GO terms are consistent with housekeeping roles in the cell (cell cycle control, cell communication, cell developmental process etc.), which are expected to be under strong purifying selection (supplementary table 10 and figs. 4–6, Supplementary Material online).

### Genes Showing Significant between-Population Divergence Are Enriched for Functional Processes Associated with Cold Adaptation

**Fig. 7.**
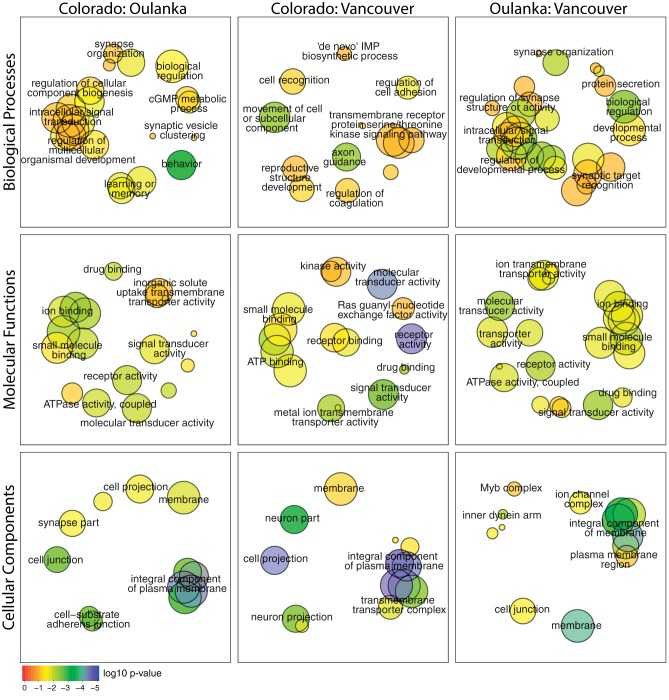
—Semantic clustering of significantly (FDR < 0.1) enriched GO-terms for genes showing significant divergence between populations of *D. montana*. Circle size corresponds to the number of genes annotated to the term in the reference database. Circle colour indicates log_10_ FDR of the GO term.

Significant outlier SNPs were found in, or within 1 kb of, 1801, 155, and 1387 genes (from pairwise comparisons between Colorado: Oulanka, Colorado: Vancouver, and Oulanka: Vancouver, respectively) (see supplementary material for detail on SNP numbers). 10 genes overlapped between all the three pairwise comparisons (supplementary fig. 7 and table 11, Supplementary Material online). Although this is a relatively small number of genes, it is significantly greater than expected by chance (*P* = 0.00013). By ranking genes by *q*-value we could identify GO terms enriched in genes with high divergence for each population comparison (Colorado: Oulanka = 74 (Biological Processes:Molecular Functions:Cellular Components = 27:29:18) (supplementary table 12, Supplementary Material online), Colorado: Vancouver = 66 (Biological Processes:Molecular Functions:Cellular Components = 19:28:19) (supplementary table 13, Supplementary Material online), Oulanka: Vancouver = 91 (Biological Processes:Molecular Functions:Cellular Components = 37:39:14) (supplementary table 14, Supplementary Material online). As with genes, there was a significant overlap of enriched GO-terms between population comparisons (*N* = 22, *P* = 1.74 × 10^−79^, supplementary fig. 7 and table 15, Supplementary Material online). Semantic clustering of GO terms (fig. 7) and functional clustering (supplementary table 16–18, Supplementary Material online) showed that the dominant terms include: membrane components, ion transport, small molecule binding, and neuron/synaptic associated terms.

**Fig. 8.**
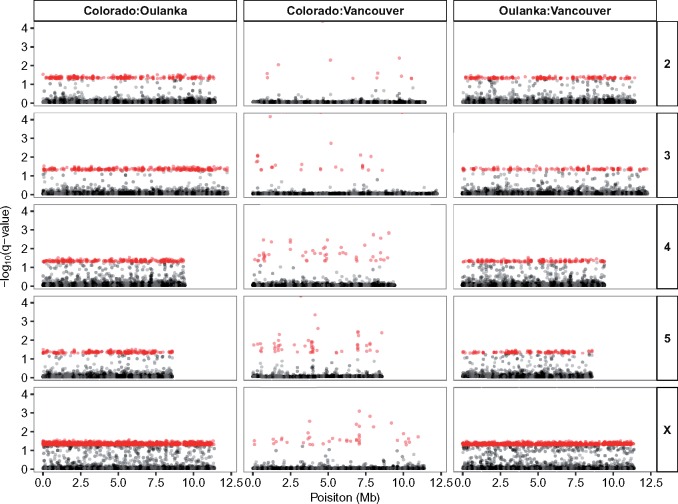
—Manhattan plot of *q*-values from the three pairwise BayeScan analyses for the SNPs on the mapped chromosomes. Red points denote SNPs which passed the 0.05 *q*-value FDR threshold. Alternating grey and black points denote different scaffolds that have been anchored to the chromosomes. The order of the mapped scaffolds is established but not their orientation.

Interestingly, outlier SNPs were not randomly distributed throughout the genome (fig. 8 and supplementary fig. 8, Supplementary Material online). There was a significant excess of outlier SNPs on the X-chromosome in all pairwise comparisons (Colorado: Oulanka—Chi-squared = 3,029.4, d.f. = 4, *P* < 0.01; Colorado: Vancouver—Chi-squared = 31.9, d.f. = 4, *P* < 0.01; Oulanka: Vancouver—Chi-squared = 2477.7, d.f. = 4, *P* < 0.01). These results held when the proportion of the total genome length of each chromosome was taken to calculate the expected numbers of SNPs.

### Genes Showing Divergence between Species and between Populations Overlap

We examined whether genes showing significant divergence between populations were the same as those showing higher rates of evolution between cold-tolerant and non-cold-tolerant species. We found 68 genes that had both an elevated rate of evolution between species and significant divergence in at least one population comparison (supplementary table 19, Supplementary Material online). This is significantly greater than we would expect by chance (Fisher’s exact test = 1.447, *P* = 0.0006) and implies that genes under divergent selection within species are also more likely to diverge between species. The functions of these genes mirror those enriched in each of the separate comparisons (transmembrane transport/ion transport [9/68], sexual reproduction [16/68], and neurological system process/neurogenesis [15/68]), implicating these genes’ involvement in similar differences in cold adaptation and reproduction between populations and species.

## Discussion

Ecological studies with *Drosophila montana* have shown that it is able to thrive at high latitudes due to a number of adaptations including the evolution of increased cold tolerance and reproductive diapause. By sequencing the genome of this species we were able to use comparative genomics to identify genes and functional processes that differ between *D. montana* and its less cold-adapted relatives. We find evidence for selection acting on neuronal, membrane-transport and ion-transport related genes at both the inter and intraspecific levels. These findings likely result from selection for an ability to overwinter under harsh environmental conditions, as these processes have clear links to both increased cold tolerance and reproductive diapause.

### Genome Assembly and Features

We assembled the *D. montana* genome using a combination of Illumina paired-end reads and mate-pair reads. We annotated 13,683 genes, which is comparable to other *Drosophila* species that have been sequenced (Clark et al. 2007). 10,898 of these genes (80%) were then assigned to a *D. virilis* ortholog, comparable to the number of orthologs identified between *D. melanogaster* and *D. simulans*. Together with the high BUSCO and CEGMA scores, this suggests that the genic component of the assembled genome is largely complete and successfully annotated.

### Inter and Intraspecific Comparisons Show Evidence for Cold Adaptation

Firstly, in the comparison between multiple *Drosophila* species, we identified 250 genes with an elevated rate of evolution between cold-tolerant and non-cold-tolerant *Drosophila* species. Interestingly, the increased rate of evolution was biased toward cold-tolerant species, with 77% of these genes showing a higher rate of evolution in these species. Secondly, we compared *D. montana* to its sequenced relative *D. virilis*. Although *D. montana* and *D. virilis* are both relatively cold-tolerant species, *D. montana* is significantly more cold-tolerant than *D. virilis* (Vesala et al. 2012), and *D. montana* is also more desiccation tolerant (Kellerman et al. 2012). In addition, unlike *D. virilis*, *D. montana* females enter reproductive diapause in late summer, which further increases their chances to survive over the cold season and produce progeny in spring (Watabe 1983). However, genes in the *D. montana* genome showed little evidence for divergent selection when compared with *D. virilis*, with most genes showing evidence for purifying selection. Finally, we compared *D. montana* populations from Oulanka, Colorado and Vancouver. These populations face quite different abiotic and biotic conditions throughout the year, and hence can be expected to vary in several traits affecting flies’ life-cycle and stress tolerances. We identified many SNPs that show significant divergence between the three populations; the number of divergent SNPs was smallest between Colorado and Vancouver populations reflecting the likely later divergence times of these populations. Although no divergent SNPs were shared between all population comparisons, when SNPs were grouped by gene, we found evidence for a significant number of overlapping genes. Divergent SNPs were overrepresented in the X chromosome which often shows elevated rates of evolution due to a combination of effects including a smaller effective population size, increased efficacy of selection in hemizygous males, and sexual antagonism. However, as some of the populations are known to differ in sexual behavior and postmating pre-zygotic reproductive barriers (Klappert et al. 2007; Jennings et al. 2014), as well as ecological adaptations, it is not possible to distinguish among the multiple possible sources of any divergent selection on X-linked SNPs.

In most of these comparisons the genes with elevated dN/dS or F_ST_ were enriched for functional processes previously demonstrated as important in cold adaptation (see below). In particular changes to membrane components and ion transport, as well as in the neurological system were heavily represented in our enrichment analyses in all comparisons. In addition, we also found enrichment of many small-molecule binding terms, but the specific terms enriched tended to be more varied across the different comparisons. Finally, several comparisons were also enriched for many reproduction-associated terms, which are unlikely to be linked to cold adaptation per se. We discuss each of these functional groups below.

### Functional Processes Enriched in Inter and Intraspecific Comparisons

#### Cellular Membranes

The composition of the cell membrane is critical for maintenance of cellular function in suboptimal temperatures (Hazel 1995; Koštál et al. 2003) with changes to cell membrane viscosity shown to be a critical component of cold acclimation in many species (Hazel 1995), including *D. melanogaster* (Cooper et al. 2014). We found enrichment of many terms associated with membrane structure (e.g., intrinsic component of membrane, integral component of membrane, plasma membrane, transmembrane region, etc.) across all our comparisons, providing further evidence for the importance of adjusting cell membrane structure to better survive in cold environments. In addition to these terms, we also found enrichment of other key processes that likely contribute to the functioning of cell membranes at low temperatures. The most important of these are functions associated with cellular ionic balance (e.g., ion channel activity, transmembrane transporter activity, calcium transport, ion binding). Many of the mechanisms involved in the maintenance of cellular ion balance are known to be temperature specific (Heitler et al. 1977; Kivivuori et al. 1990). Failure to maintain the ionic balance of cells leads to metabolic perturbations which can cause a wide range of negative consequences, including cellular damage and even death (Hochachka 1986; Koštál et al. 2004). One class of cells particularly affected by low temperature are neurons (Montgomery and Macdonald 1990; Janssen 1992; Robertson and Money 2012) which are particularly susceptible to cold injury (Hochachka and Somero 2002). In line with this we also found enrichment of several terms related to neuron function (cell projection membrane, dendrite membrane, signal transducer activity, etc.). Finally, we observed that membrane, ion transport, and neuronal terms often functionally clustered together, showing that changes to each of these functions are in fact interrelated. Taken together this suggests that the adjustment of cell membranes for increased cold tolerance is complex, requiring changes to many genes to improve cellular functioning at low temperatures.

#### Small-Molecule Binding

We observed enrichment of many small-molecule binding terms (small-molecule binding, ATP-binding, kinase, nucleotide-binding, nucleotide phosphate-binding, carbohydrate derivative binding, ribonucleotide binding, anion binding, etc.), both in the population and in the multi-species comparisons. At low temperatures the activity levels of many reactions are reduced meaning that during cold adaptation there is selection to adjust chemical reactions to work better in cold environments (Margesin 2017). In particular ATP-binding and associated terms were enriched in most of our comparisons suggesting that adjustments to ATP-binding may be particularly important for cold adaptation. This finding is supported by the fact that low temperatures adversely affect ATP metabolism across a broad range of taxa (Napolitano and Shain 2004; Morrison and Shain 2008), including freeze-tolerant species like the terrestrial earthworm (*Enchytraeus albidus*) that are able to survive winters in a frozen state (Boer et al. 2017).

#### Reproduction

Genes involved in reproduction typically show faster rates of divergence than other genes (Swanson and Vacquier 2002; Clark et al. 2006). Consistent with this we find reproductive-associated terms (male courtship behavior, single organism reproductive process, reproductive process) are enriched at each comparison level. Different species of *Drosophila* (including *D. montana* and *D. virilis*) are known to vary for a number of reproductive traits and so this finding is not too surprising. Interestingly, the only pairwise population comparison that shows enrichment for reproductive-associated terms (reproductive structure development, gonad development) is between Colorado and Vancouver. Although all populations show some evidence of reproductive isolation, crosses between Colorado and Vancouver showed the highest proportion of nondeveloping eggs (Jennings et al. 2014). Moreover, although the exact cause of nondeveloping eggs is unknown, one possibility is that it could be due to a negative interaction between sperm and the female reproductive tract. Some support for this idea comes from examining the top differentiated genes between Colorado and Vancouver which include the transcription factor *ken and barbie* which has a major role in the development of genitalia of *D. melanogaster* (Lukacsovich et al. 2003).

### Functional Processes Enriched in Specific-Comparisons

Although we observe many terms related to cold tolerance common to each of our comparisons (described above), we also observe enrichment of several other functional processes which are restricted to one or two of our comparisons. Of these, two (Olfaction and cuticular processes) are of particular interest due to their potential link to cold adaptation and are discussed below.

#### Cuticular Related Processes

Cuticular and chitin related processes show an extensive enrichment in genes showing elevated dN/dS between *D. montana* and *D. virilis*, but not in the multi-species or population comparisons. Changes to the cuticle are linked to increased cold and desiccation tolerance in insects (Gibbs 2002; Dennis et al. 2015) and in particular to enhancing the stress resistance of the cuticle during diapause (Li and Denlinger 2009; Benoit 2010). This is particularly interesting as *D. montana*, unlike *D. virilis*, has a reproductive diapause meaning the changes we observe in cuticular related genes may have resulted from selection for increased stress resistance to help *D. montana* successfully overwinter. This idea is consistent with the fact that cuticular related processes are only found in the *D. montana–D. virilis* comparison, as this is the only one of our comparisons that directly compares non-diapausing and diapausing capable groups.

#### Olfaction

*Drosophila* flies have various kinds of olfaction-driven behaviors including the location of food and mates (Amrein 2004; Libert et al. 2007) and the genomic repertoire of olfactory loci is correlated with environmental variation (Gardiner et al. 2008). A cold environment may affect the perception of olfactory signals as the detection of odorants at low temperatures is more difficult due to the reduced concentration of olfactory cues in the air. Previous work in *D. melanogaster* has shown that the sensitivity of the olfactory system increases in response to cold temperature (e.g., Dalton 2000), and that this change is accompanied by a change in expression in olfactory genes (Riveron et al. 2009, 2013). Since both sexual and nonsexual olfactory signals are likely to be affected by colder temperatures, we hypothesize that the changes in olfaction-related genes we observe in the present study are a product of adaptation to living in a colder environment as well as of sexual selection to distinguish conspecific flies from the heterospecific ones. Olfaction related terms were enriched in both species-level comparisons, but not population comparisons.

### Population and Species Divergence at Common Loci

Phenotypic variation in similar traits between and within species may or may not arise from the same genes even when selection processes are similar (Wittkopp et al. 2009). Here, we find that genes which show divergence between populations were also more likely to show elevated differences between species. The functions of these genes mirror those enriched in each of the separate comparisons (transmembrane transport/ion transport sexual reproduction and neurological system processes), implicating these genes’ involvement in similar differences in cold adaptation and reproduction between populations and species. Although any of these genes may be important in cold adaptation, one gene in particular, *Task6*, stands out as an interesting candidate. *Task6* encodes a subunit of two-pore domain potassium (K2P) channels, which are important in setting the membrane potential and input resistance of neurons in Drosophila (Döring et al. 2006). Temperature impacts a cell’s ability to maintain ionic balance, and in particular a loss of potassium ion balance has been shown to cause membrane depolarization, induction of chill-coma, and cell death (Andersen et al. 2015; Andersen et al. 2017). As such the changes we observe in *Task6* may be involved in thermal adaptation of species and populations.

### Conclusion

*D* *rosophila* *montana* is an exceptional species of *Drosophila* in terms of cold adaptation, as well as a species used for studies of behavioral variation and reproductive isolation. Here, we report the first description of its genome. Although there are few strong signals of divergent selection on coding sequence variation, especially with its closest available relative, contrasts between cold-adapted species and intraspecific population sequencing suggest that the genome contains a clear signal of selection for cold tolerance. We identify many genes potentially important in adaptation and speciation in this ecological specialist species.

## Supplementary Material

Supplementary Data

## References

[evy147-B1] Amrein H. 2004 Pheromone perception and behavior in Drosophila. Curr Opin Neurobiol. 14(4):435–442. 15321064 10.1016/j.conb.2004.07.008

[evy147-B2] Andersen JL, et al 2015 How to assess Drosophila cold tolerance: chill coma temperature and lower lethal temperature are the best predictors of cold distribution limits. Funct Ecol. 29(1):55–65.

[evy147-B3] Andersen MK, Jensen SO, Overgaard J. 2017 Physiological correlates of chill susceptibility in Lepidoptera. J Insect Physiol. 98:317–326. 28188725 10.1016/j.jinsphys.2017.02.002

[evy147-B4] Angelis K, Dos Reis M, Yang Z. 2014 Bayesian estimation of nonsynonymous/synonymous rate ratios for pairwise sequence comparisons. Mol Biol Evol. 31(7):1902–1913. 24748652 10.1093/molbev/msu142 PMC4069626

[evy147-B5] Benoit JB. 2010 Water management by dormant insects: comparisons between dehydration resistance during summer aestivation and winter diapause In: Aestivation. progress in molecular and subcellular biology. Berlin, Heidelberg: Springer p. 209–229. 10.1007/978-3-642-02421-4_10 20069411

[evy147-B6] Boer TE, Roelofs D, Vooijs R, Holmstrup M, Amorim MJ. 2017 Population-specific transcriptional differences associated with freeze tolerance in a terrestrial worm. Ecol Evol. 8: 3774–3786. 10.1002/ece3.3602 PMC5901168 29686857

[evy147-B7] Bolger AM, Lohse M, Usadel B. 2014 Trimmomatic: a flexible trimmer for Illumina sequence data. Bioinformatics 30(15):2114–2120. 24695404 10.1093/bioinformatics/btu170 PMC4103590

[evy147-B8] Broman KW, Wu H, Sen S, Churchill GA. 2003 R/qtl: QTL mapping in experimental crosses. Bioinformatics 19(7):889–890. 12724300 10.1093/bioinformatics/btg112

[evy147-B9] Buffalo V. 2014 Scythe. Available from: https://github.com/vsbuffalo/scythe.

[evy147-B10] Chen C, et al 2016 Genomic analyses reveal demographic history and temperate adaptation of the newly discovered honey bee subspecies *Apis mellifera sinisxinyuan* n. ssp. Mol Biol E 33:1337–1348. 10.1093/molbev/msw017 PMC4839221 26823447

[evy147-B11] Clark AG, et al 2007 Evolution of genes and genomes on the Drosophila phylogeny. Nature 450:203–218. 17994087 10.1038/nature06341

[evy147-B12] Clark NL, Aagaard JE, Swanson WJ. 2006 Evolution of reproductive proteins from animals and plants. Reproduction 131:11–22. 16388004 10.1530/rep.1.00357

[evy147-B13] Cooper BS, Hammad LA, Montooth KL. 2014 Thermal adaptation of cellular membranes in natural populations of Drosophila melanogaster. Funct Ecol. 28:886–894. 25382893 10.1111/1365-2435.12264 PMC4219941

[evy147-B14] Cruickshank TE, Hahn MW. 2014 Reanalysis suggests that genomic islands of speciation are due to reduced diversity, not reduced gene flow. Mol Ecol. 23:3133–3157. 24845075 10.1111/mec.12796

[evy147-B15] Dalton P. 2000 Psychophysical and behavioral characteristics of olfactory adaptation. Chem Senses 25(4):487–492. 10944515 10.1093/chemse/25.4.487

[evy147-B16] Dennis AB, Dunning LT, Sinclair BJ, Buckley TR. 2015 Parallel molecular routes to cold adaptation in eight genera of New Zealand stick insects. Sci Rep. 5:13965. 26355841 10.1038/srep13965 PMC4564816

[evy147-B17] Döring F, Scholz H, Kühnlein RP, Karschin A, Wischmeyer E. 2006 Novel Drosophila two-pore domain K+ channels: rescue of channel function by heteromeric assembly. Eur J Neurosci. 24(8):2264–2274. 17074048 10.1111/j.1460-9568.2006.05102.x

[evy147-B18] Eden E, Navon R, Steinfeld I, Lipson D, Yakhini Z. 2009 GOrilla: a tool for discovery and visualization of enriched GO terms in ranked gene lists. BMC Bioinformatics 10:48. 19192299 10.1186/1471-2105-10-48 PMC2644678

[evy147-B19] Ellegren H. 2008 Comparative genomics and the study of evolution by natural selection. Mol Ecol. 17(21):4586–4596. 19140982 10.1111/j.1365-294X.2008.03954.x

[evy147-B20] Fischer MC, Foll M, Excoffier L, Heckel G. 2011 Enhanced AFLP genome scans detect local adaptation in high-altitude populations of a small rodent (*Microtus arvalis*). Mol Ecol. 20(7):1450–1462. 21352386 10.1111/j.1365-294X.2011.05015.x

[evy147-B21] Foll M, Fischer MC, Heckel G, Excoffier L. 2010 Estimating population structure from AFLP amplification intensity. Mol Ecol. 19(21):4638–4647. 20874760 10.1111/j.1365-294X.2010.04820.x

[evy147-B22] Foll M, Gaggiotti O. 2008 A genome-scan method to identify selected loci appropriate for both dominant and codominant markers: a bayesian perspective. Genetics 180(2):977–993. 18780740 10.1534/genetics.108.092221 PMC2567396

[evy147-B23] Foll M, Gaggiotti OE, Daub JT, Vatsiou A, Excoffier L. 2014 Widespread signals of convergent adaptation to high altitude in Asia and America. Am J Hum Genet. 95(4):394–407. 25262650 10.1016/j.ajhg.2014.09.002 PMC4185124

[evy147-B24] Gardiner A, Barker D, Butlin RK, Jordan WC, Ritchie MG. 2008 Drosophila chemoreceptor gene evolution: selection, specialization and genome size. Mol Ecol. 17(7):1648–1657. 18371013 10.1111/j.1365-294X.2008.03713.x

[evy147-B25] Gibbs AG. 2002 Lipid melting and cuticular permeability: new insights into an old problem. J Insect Physiol. 48(4):391–400. 12770088 10.1016/s0022-1910(02)00059-8

[evy147-B26] Haerty W, et al 2007 Evolution in the fast lane: rapidly evolving sex-related genes in *Drosophila*. Genetics 177(3):1321–1335. 18039869 10.1534/genetics.107.078865 PMC2147986

[evy147-B27] Hazel JR. 1995 Thermal adaptation in biological membranes—is homeoviscous adaptation the explanation? Annu Rev Physiol. 57:19–42. 7778864 10.1146/annurev.ph.57.030195.000315

[evy147-B28] Heitler WJ, Goodman CS, Fraserrowell CH. 1977 Effects of temperature on threshold of identified neurons in locust. J Comp Physiol. 117(2):163–182.

[evy147-B29] Hochachka PW. 1986 Defense strategies against hypoxia and hypothermia. Science 231(4735):234–241. 2417316 10.1126/science.2417316

[evy147-B30] Hochachka PW, Somero GN. 2002 Temperature In: Biochemical adaptation: mechanism and process in physiological evolution. New York: Oxford University Press p. 290–449.

[evy147-B31] Holt C, Yandell M. 2011 MAKER2: an annotation pipeline and genome-database management tool for second-generation genome projects. BMC Bioinformatics 12:491. 22192575 10.1186/1471-2105-12-491 PMC3280279

[evy147-B32] Huang DW, Sherman BT, Lempicki RA. 2009 Bioinformatics enrichment tools: paths toward the comprehensive functional analysis of large gene lists. Nucleic Acids Res. 37(1):1–13. 19033363 10.1093/nar/gkn923 PMC2615629

[evy147-B33] Huang DW, Sherman BT, Lempicki RA. 2009 Systematic and integrative analysis of large gene lists using DAVID bioinformatics resources. Nat Protoc. 4(1):44–57. 19131956 10.1038/nprot.2008.211

[evy147-B34] Janssen R. 1992 Thermal influences on nervous system function. Neurosci Biobehav Rev. 16(3):399–413. 1528527 10.1016/s0149-7634(05)80209-x

[evy147-B35] Jennings JH, Mazzi D, Ritchie MG, Hoikkala A. 2011 Sexual and postmating reproductive isolation between allopatric *Drosophila montana* populations suggest speciation potential. BMC Evol Biol. 11:68. 21396136 10.1186/1471-2148-11-68 PMC3065424

[evy147-B36] Jennings JH, Snook RR, Hoikkala A. 2014 Reproductive isolation among allopatric *Drosophila montana* populations. Evolution 68(11):3095–3108. 25302639 10.1111/evo.12535

[evy147-B37] Joshi NA, Fass JN. 2011 Sickle: a sliding-window, adaptive, quality-based trimming tool for FastQ files. Available from: https://github.com/najoshi/sickle.

[evy147-B38] Kankare M, Parker DJ, Merisalo M, Salminen TS, Hoikkala A. 2016 Transcriptional differences between diapausing and non-diapausing *D. montana* females reared under the same photoperiod and temperature. PLoS One 11(8):e0161852. 27571415 10.1371/journal.pone.0161852 PMC5003386

[evy147-B39] Kauranen H, Ala-Honkola O, Kankare M, Hoikkala A. 2016 Circadian clock of *Drosophila montana* is adapted to high variation in summer day lengths and temperatures prevailing at high latitudes. J Insect Physiol. 89:9–18. 26993661 10.1016/j.jinsphys.2016.03.005

[evy147-B40] Kauranen H, et al 2012 Flies in the north: locomotor behavior and clock neuron organization of *Drosophila montana*. J Biol Rhythms 27(5):377–387. 23010660 10.1177/0748730412455916

[evy147-B41] Kellermann V, et al 2012 Phylogenetic constraints in key functional traits behind species’ climate niches: patterns of desiccation and cold resistance across 95 *Drosophila* species. Evolution 66:3377–3389. 23106704 10.1111/j.1558-5646.2012.01685.x

[evy147-B42] Kivivuori L, Lehti S, Lagerspetz KYH. 1990 Effect of temperature-acclimation on thermal-dependence and hysteresis of the resting membrane-potential of the stretch-receptor neuron in crayfish *Astacus astacus* (L). J Therm Biol. 15:9–14.

[evy147-B43] Klappert K, Mazzi D, Hoikkala A, Ritchie MG. 2007 Male courtship song and female preference variation between phylogeographically distinct populations of *Drosophila montana*. Evolution 61:1481–1488. 17542854 10.1111/j.1558-5646.2007.00125.x

[evy147-B44] Kofler R, Pandey RV, Schlötterer C. 2011 PoPoolation2: identifying differentiation between populations using sequencing of pooled DNA samples (Pool-Seq). Bioinformatics 27:3435–3436. 22025480 10.1093/bioinformatics/btr589 PMC3232374

[evy147-B45] Koštál V, Berková P, Šimek P. 2003 Remodelling of membrane phospholipids during transition to diapause and cold-acclimation in the larvae of *Chymomyza costata* (Drosophilidae). Comp Biochem Physiol B Biochem Mol Biol. 135(3):407–419. 12831761 10.1016/s1096-4959(03)00117-9

[evy147-B46] Koštál V, Vambera J, Bastl J. 2004 On the nature of pre-freeze mortality in insects: water balance, ion homeostasis and energy charge in the adults of *Pyrrhocoris apterus*. J Exp Biol. 207(Pt 9):1509–1521. 15037645 10.1242/jeb.00923

[evy147-B47] Lakovaara S. 1969 Malt as a culture medium for Drosophila species. Drosoph Inf Serv. 44:128.

[evy147-B48] Lankinen P, Tyukmaeva VI, Hoikkala A. 2013 Northern *Drosophila montana* flies show variation both within and between cline populations in the critical day length evoking reproductive diapause. J Insect Physiol. 59(8):745–751. 23702203 10.1016/j.jinsphys.2013.05.006

[evy147-B49] Li A, Denlinger DL. 2009 Pupal cuticle protein is abundant during early adult diapause in the mosquito *Culex pipiens*. J Med Entomol. 46(6):1382–1386. 19960684 10.1603/033.046.0618

[evy147-B50] Li H, Durbin R. 2009 Fast and accurate short read alignment with Burrows–Wheeler transform. Bioinformatics 25(14):1754–1760. 19451168 10.1093/bioinformatics/btp324 PMC2705234

[evy147-B51] Li H, et al 2009 The sequence alignment/map format and SAMtools. Bioinformatics 25(16):2078–2079. 19505943 10.1093/bioinformatics/btp352 PMC2723002

[evy147-B52] Libert S, et al 2007 Regulation of Drosophila life span by olfaction and food-derived odors. Science 315(5815):1133–1137. 17272684 10.1126/science.1136610

[evy147-B53] Löytynoja A, Goldman N. 2005 An algorithm for progressive multiple alignment of sequences with insertions. Proc Natl Acad Sci USA. 102(30):10557–10562. 16000407 10.1073/pnas.0409137102 PMC1180752

[evy147-B54] Lukacsovich T, et al 2003 The *ken and barbie* gene encoding a putative transcription factor with a BTB domain and three zinc finger motifs functions in terminalia development of Drosophila. Arch Insect Biochem Physiol. 54(2):77–94. 14518006 10.1002/arch.10105

[evy147-B55] MacMillan HA, et al 2015 Parallel ionoregulatory adjustments underlie phenotypic plasticity and evolution of Drosophila cold tolerance. J Exp Biol. 218(Pt 3):423–432. 25524989 10.1242/jeb.115790

[evy147-B56] Margesin R. 2017 Psychrophiles: from biodiversity to biotechnology. London, UK: Springer.

[evy147-B57] Matzkin LM, Watts TD, Bitler BG, Machado CA, Markow TA. 2006 Functional genomics of cactus host shifts in *Drosophila mojavensis*. Mol Ecol. 15(14):4635–4643. 17107489 10.1111/j.1365-294X.2006.03102.x

[evy147-B58] McKenna DD, et al 2016 Genome of the Asian longhorned beetle (*Anoplophora glabripennis*), a globally significant invasive species, reveals key functional and evolutionary innovations at the beetle–plant interface. Genome Biol. 17(1):227. 27832824 10.1186/s13059-016-1088-8 PMC5105290

[evy147-B59] Menegazzi P, et al 2017 Adaptation of circadian neuronal network to photoperiod in high-latitude European drosophilids. Curr Biol. 27(6):833–839. 28262491 10.1016/j.cub.2017.01.036

[evy147-B60] Mirol PM, et al 2007 Phylogeographic patterns in *Drosophila montana*. Mol Ecol. 16(5):1085–1097. 17305862 10.1111/j.1365-294X.2006.03215.x

[evy147-B61] Montgomery JC, Macdonald JA. 1990 Effects of temperature on nervous system: implications for behavioral performance. Am J Physiol. 259(2):R191–R196. 2201212 10.1152/ajpregu.1990.259.2.R191

[evy147-B62] Morrison BA, Shain DH. 2008 An AMP nucleosidase gene knockout in *Escherichia coli* elevates intracellular ATP levels and increases cold tolerance. Biol Lett. 4(1):53–56. 18029299 10.1098/rsbl.2007.0432 PMC2412920

[evy147-B63] Napolitano MJ, Shain DH. 2004 Four kingdoms on glacier ice: convergent energetic processes boost energy levels as temperatures fall. Proc R Soc Lond B Biol Sci. 271(Suppl 5):S273–S276. 10.1098/rsbl.2004.0180 PMC1810069 15503992

[evy147-B64] Noor MA, Bennett SM. 2009 Islands of speciation or mirages in the desert? Examining the role of restricted recombination in maintaining species. Heredity 103(6):439. 19920849 10.1038/hdy.2009.151 PMC2809014

[evy147-B65] Nosil P, Funk DJ, Ortiz-Barrientos D. 2009 Divergent selection and heterogeneous genomic divergence. Mol Ecol. 18(3):375–402. 19143936 10.1111/j.1365-294X.2008.03946.x

[evy147-B66] Ostrega MS. 1985 Restriction endonuclease analysis of the relatedness of *D. montana* and *D. virilis* lines. Drosoph Inf Serv. 61:132–133.

[evy147-B67] Parker DJ, Ritchie MG, Kankare M. 2016 Preparing for Winter: the Transcriptomic Response Associated with Different Day Lengths in *Drosophila montana*. G3 116:027870. 10.1534/g3.116.027870 PMC4856088 26976440

[evy147-B68] Parker DJ, et al 2015 How consistent are the transcriptome changes associated with cold acclimation in two species of the *Drosophila virilis* group? Heredity 115(1):13–21. 25669607 10.1038/hdy.2015.6 PMC4815502

[evy147-B69] Parra G, Bradnam K, Korf I. 2007 CEGMA: a pipeline to accurately annotate core genes in eukaryotic genomes. Bioinformatics 23(9):1061–1067. 17332020 10.1093/bioinformatics/btm071

[evy147-B70] Parra G, Bradnam K, Ning ZM, Keane T, Korf I. 2009 Assessing the gene space in draft genomes. Nucleic Acids Res. 37(1):289–297. 19042974 10.1093/nar/gkn916 PMC2615622

[evy147-B71] Paterson T, Law A. 2011 Genotypechecker: an interactive tool for checking the inheritance consistency of genotyped pedigrees. Anim Genet. 42(5):560–562. 21906109 10.1111/j.1365-2052.2011.02183.x

[evy147-B72] Pérez-Figueroa A, García-Pereira MJ, Saura M, Rolán-Alvarez E, Caballero A. 2010 Comparing three different methods to detect selective loci using dominant markers. J Evol Biol. 23(10):2267–2276. 20796133 10.1111/j.1420-9101.2010.02093.x

[evy147-B73] Radwan J, Babik W. 2012 The genomics of adaptation. Proc R Soc Lond B Biol Sci. 279(1749):5024–5028. 10.1098/rspb.2012.2322 PMC3497254 23097510

[evy147-B74] Ravinet M, et al 2017 Interpreting the genomic landscape of speciation: a road map for finding barriers to gene flow. J Evol Biol. 30(8):1450–1477. 28786193 10.1111/jeb.13047

[evy147-B75] Riveron J, Boto T, Alcorta E. 2009 The effect of environmental temperature on olfactory perception in *Drosophila melanogaster*. J Insect Physiol. 55(10):943–951. 19559705 10.1016/j.jinsphys.2009.06.009

[evy147-B76] Riveron J, Boto T, Alcorta E. 2013 Transcriptional basis of the acclimation to high environmental temperature at the olfactory receptor organs of *Drosophila melanogaster*. BMC Genomics 14:259. 23590196 10.1186/1471-2164-14-259 PMC3653700

[evy147-B77] Robertson RM, Money TG. 2012 Temperature and neuronal circuit function: compensation, tuning and tolerance. Curr Opin Neurobiol. 22(4):724–734. 22326854 10.1016/j.conb.2012.01.008

[evy147-B78] Sackton TB, et al 2007 Dynamic evolution of the innate immune system in Drosophila. Nat Genet. 39(12):1461–1468. 17987029 10.1038/ng.2007.60

[evy147-B79] Salminen TS, et al 2015 Seasonal gene expression kinetics between diapause phases in *Drosophila virilis* group species and overwintering differences between diapausing and non-diapausing females. Sci Rep. 5(1): 11197. 26063442 10.1038/srep11197 PMC4463020

[evy147-B80] Schäfer MA, et al 2010 A microsatellite linkage map for *Drosophila montana* shows large variation in recombination rates, and a courtship song trait maps to an area of low recombination. J Evol Biol. 23(3):518–527. 20040000 10.1111/j.1420-9101.2009.01916.x

[evy147-B81] Schmieder R, Edwards R. 2011 Fast identification and removal of sequence contamination from genomic and metagenomic datasets. PLoS One 6(3):e17288. 21408061 10.1371/journal.pone.0017288 PMC3052304

[evy147-B82] Simão FA, Waterhouse RM, Ioannidis P, Kriventseva EV, Zdobnov EM. 2015 BUSCO: assessing genome assembly and annotation completeness with single-copy orthologs. Bioinformatics 31(19):3210–3212. 26059717 10.1093/bioinformatics/btv351

[evy147-B83] Smadja CM, Butlin RK. 2011 A framework for comparing processes of speciation in the presence of gene flow. Mol Ecol. 20(24):5123–5140. 22066935 10.1111/j.1365-294X.2011.05350.x

[evy147-B84] Smith G, et al 2013 Transcriptome-wide expression variation associated with environmental plasticity and mating success in cactophilic *Drosophila mojavensis*. Evolution 67(7):1950–1963. 23815652 10.1111/evo.12082

[evy147-B85] Spicer GS, Bell CD. 2002 Molecular phylogeny of the *Drosophila virilis* species group (Diptera: drosophilidae) inferred from mitochondrial 12S and 16S ribosomal RNA genes. Ann Entomol Soc Am. 95:156–161.

[evy147-B86] Supek F, Bošnjak M, Škunca N, Šmuc T. 2011 REVIGO summarizes and visualizes long lists of gene ontology terms. PLoS One 6(7):e21800. 21789182 10.1371/journal.pone.0021800 PMC3138752

[evy147-B87] Swanson WJ, Vacquier VD. 2002 Reproductive protein evolution. Annu Rev Ecol Syst. 33(1):161–179.

[evy147-B88] Tapanainen R, Parker DJ, Kankare M. 2018 Photosensitive alternative splicing of the circadian clock gene timeless is population specific in a cold-adapted fly, *Drosophila montana*. G3 8:1291–1297. 29472309 10.1534/g3.118.200050 PMC5873918

[evy147-B89] Terhzaz S, et al 2015 Insect capa neuropeptides impact desiccation and cold tolerance. Proc Natl Acad Sci USA. 112(9):2882. 25730885 10.1073/pnas.1501518112 PMC4352776

[evy147-B90] Throckmorton LH. 1982 The virilis species group In: Ashburner M, Carson HL, Thompson JN, editors. Genetics and Biology of Drosophila. Vol. 3b London, UK: Academic Press p. 227–296.

[evy147-B91] Tweedie S, et al 2009 FlyBase: enhancing *Drosophila* gene ontology annotations. Nucleic Acids Res. 37(Database):D555–D559. 18948289 10.1093/nar/gkn788 PMC2686450

[evy147-B92] Tyukmaeva VI, Salminen TS, Kankare M, Knott KE, Hoikkala A. 2011 Adaptation to a seasonally varying environment: a strong latitudinal cline in reproductive diapause combined with high gene flow in *Drosophila montana*. Ecol Evol. 1(2):160–168. 22393492 10.1002/ece3.14 PMC3287301

[evy147-B93] Tyukmaeva VI, et al 2015 Localization of quantitative trait loci for diapause and other photoperiodically regulated life history traits important in adaptation to seasonally varying environments. Mol Ecol. 24(11):2809–2819. 25877951 10.1111/mec.13202

[evy147-B94] Vesala L, Hoikkala A. 2011 Effects of photoperiodically induced reproductive diapause and cold hardening on the cold tolerance of *Drosophila montana*. J Insect Physiol. 57(1):46–51. 20932841 10.1016/j.jinsphys.2010.09.007

[evy147-B95] Vesala L, Salminen TS, Laiho A, Hoikkala A, Kankare M. 2012 Cold tolerance and cold-induced modulation of gene expression in two *Drosophila* virilis group species with different distributions. Insect Mol Biol. 21(1):107–118. 22122733 10.1111/j.1365-2583.2011.01119.x

[evy147-B96] Vigoder FM, et al 2016 Inducing Cold-Sensitivity in the Frigophilic Fly Drosophila montana by RNAi. PLoS One 11(11):e0165724. 27832122 10.1371/journal.pone.0165724 PMC5104470

[evy147-B97] Vilas A, Pérez-Figueroa A, Caballero A. 2012 A simulation study on the performance of differentiation-based methods to detect selected loci using linked neutral markers. J Evol Biol. 25(7):1364–1376. 22551238 10.1111/j.1420-9101.2012.02526.x

[evy147-B98] Villemereuil P, Frichot É, Bazin É, François O, Gaggiotti OE. 2014 Genome scan methods against more complex models: when and how much should we trust them? Mol Ecol. 23(8):2006–2019. 24611968 10.1111/mec.12705

[evy147-B99] Watabe H. 1983 Photoperiodic responses in the *Drosophila virilis* species group (Diptera, Drosophilidae) from Japan. Kontyo 51:628–634.

[evy147-B100] Wittkopp PJ, et al 2009 Intraspecific polymorphism to interspecific divergence: genetics of pigmentation in Drosophila. Science 326(5952):540–544. 19900891 10.1126/science.1176980

[evy147-B101] Wolf JBW, Ellegren H. 2017 Making sense of genomic islands of differentiation in light of speciation. Nat Rev Genet. 18(2):87–100. 27840429 10.1038/nrg.2016.133

[evy147-B102] Yang ZH. 1997 PAML: a program package for phylogenetic analysis by maximum likelihood. Comput Appl Biosci. 13(5):555–556. 9367129 10.1093/bioinformatics/13.5.555

[evy147-B103] Yang ZH, Bielawski JP. 2000 Statistical methods for detecting molecular adaptation. Trends Ecol Evol. 15(12):496–503. 11114436 10.1016/S0169-5347(00)01994-7 PMC7134603

[evy147-B104] Zhu Y, Bergland AO, González J, Petrov DA. 2012 Empirical validation of pooled whole genome population re-sequencing in *Drosophila melanogaster*. PLoS One 7(7):e41901. 22848651 10.1371/journal.pone.0041901 PMC3406057

