# Supplementary material for: Inter and Intraspecific Genomic Divergence in Drosophila montana Shows Evidence for Cold Adaptation
Source: Genome Biol Evol. 2018 Jul 13;10(8):2086–101. doi: 10.1093/gbe/evy147 (PMC6107330; doi:10.1093/gbe/evy147)
Supplement: Supplementary Data [file evy147_suppl.zip › montana_genome_v8.0_supp_methods.doc]

**SUPPLEMENTAL MATERIALS**

**Supplemental methods**

**Genome Annotation**

We used the Maker2 pipeline (Holt and Yandell 2011) which first masks putative repeats within a genome and then uses information from *ab initio* gene predictors, and gene evidence (from proteins and RNA) to generate gene predictions. Maker2 uses RepeatMasker (v. 4.0.1) (Smit et al. 2015) to mask repeats from the Repbase database (downloaded on 8^th^ April 2013) (Jurka et al. 2005). We also used the default transposable element (TE) sequences from Maker2 to mask TEs.

Maker2 was run in a 2-step iterative process. In the first we provided a trained SNAP model, transcript information, and protein information to Maker2 as described below. To generate a trained SNAP (v. 2006-07-28) gene model, we used the annotation generated from running CEGMA on our genome (above) as a training set. To provide transcript information we used RNA-seq reads from previous studies investigating cold acclimation (Parker et al. 2015) and diapause (Parker et al. 2016) (Accession numbers: SRR2910689, SRR2910692, SRR2910693, SRR2910695, SRR2910698, SRR2910701, SRR2910702, SRR2910703, SRR2910704, SRR2910705, SRR1501259, SRR1501260, SRR1501261, SRR1501262, SRR1501263, SRR1501264). These reads were trimmed using SOLiD TRIM (options: -p 4 -q 25 -y on -e 3 -d 10) (Sasson and Michael 2010) and the reads that passed this filter were then error corrected using SOLiD Accuracy Enhancer Tools (SAET) to reduce the amount of color calling errors, or erroneous bases, in the sequence. These reads were then mapped to the genome assembly with TopHat2 (Kim et al. 2013) (v. 2.0.10) (options: --read-mismatches 3 --read-gap-length 3 --read-edit-dist 3) and Cufflinks (Trapnell et al. 2010) (v. 2.1.1) (default options) to produce an annotation (gtf) file. This file was converted to GFF3 format using the cufflinks2gff3 script so it could be passed to Maker2. Protein homology evidence was provided using protein sequences from FlyBase for *D. virilis* (r1.2) and *D. melanogaster* (r5.56) and Maker2 was run using default options except for est2genome=1, protein2genome=1. The annotations from this round were then used to train the SNAP and AUGUSTUS (v. 2.5.5) gene predictors and Maker2 was run again using the protein and transcript evidence as before, the updated SNAP and AUGUSTUS gene models and default options except est2genome=0, protein2genome=0. Maker2 calculates the Annotation Edit Distance (AED) for its annotations which quantifies how well predictors match evidence and we used this score to select annotations with AED < 1.

We obtained 13,683 predicted gene models with a mean coding region length of 1,461 bp. RBBH identified 10898 orthologs with *D. virilis*. Using the FlyBase gene numbers for *D. virilis* for these we then obtained a set of 5619 one-to-one orthologs between *D. montana*, *D. virilis*, *D. melanogaster*, *D. sechellia*, *D. simulans*, *D. erecta*, *D. yakuba*, *D. ananassae*, *D. persimilis*, *D. pseudoobscura*, *D. willistoni*, *D. mojavensis*, and *D. grimshawi*.

**Supplemental results**

**Between-population SNPs**

A total of 4,802,266 SNPs were analysed. Of these, 1,669,212 (35%) could be placed on the main linkage groups. Using an FDR threshold of q < 0.05, a total of 29,556 SNPs were identified as outliers (10,113 could be placed on linkage groups, **Supplementary Figures 10-12**). The outlier SNPs that could be placed on linkage groups were not randomly distributed throughout the genome (**Supplementary Figures 11,12**) (Chi-squared = 9087.7, df = 4, p < 0.001). When the proportion of the total genome length of each chromosome was used to calculate the expected numbers of SNPs, there was still a significant deviation (Chi-squared = 9541.1, df = 4, p < 0.001). There were more observed outlier SNPs on the X-chromosome (5,840) than expected (1,977). However, these results should be taken with caution because the SNP counts on each chromosome are not completely independent due to LD between SNPs (**Supplementary Figure 12**). In the three pairwise analyses, results were qualitatively similar. The numbers of SNPs in each comparison along with the number of outlier SNPs are presented in (**Supplementary Table 20)**.

There were fewer polymorphic SNPs between the Colorado and Vancouver populations and there were also fewer outlier SNPs in this comparison (**Supplementary Table 20**, **Supplementary Figure 13**). As above, the outlier SNPs are not randomly distributed throughout the genome (**Fig. 7 and Supplementary Figure 8**). There was a significant excess of outlier SNPs on the X-chromosome in all pairwise comparisons (Colorado: Oulanka – Chi-squared = 3,029.4, d.f. = 4, p < 0.01; Colorado: Vancouver - Chi-squared = 31.9, d.f. = 4, p < 0.01; Oulanka: Vancouver - Chi-squared = 2477.7, d.f. = 4, p < 0.01). These results held when the proportion of the total genome length of each chromosome was taken to calculate the expected numbers of SNPs. There were no shared outlier SNPs between all three pairwise comparisons but there were some shared outlier SNPs between pairs of population comparisons and the majority of outlier SNPs were private to each comparison (**Supplementary Figure 7**).

To examine patterns of divergence at the gene level we considered a gene to be differentiated if it contained a significantly differentiated SNP (with 1kb) (**Supplementary Table 2**).

**Supplementary Table 20 |** The total number of SNPs and the number of outlier SNPs from pairwise BayeScan analyses. The q-value threshold for calling outlier SNPs was 0.05.

| Comparison | N SNPs | N Outlier SNPs |
| --- | --- | --- |
| Colorado:Oulanka | 4,347,626 | 8,570 |
| Colorado:Vancouver | 3,921,665 | 551 |
| Oulanka:Vancouver | 4,140,171 | 6,097 |

**References**

Holt C, Yandell M. 2011. MAKER2: an annotation pipeline and genome-database management tool for second-generation genome projects. BMC Bioinformatics 12:491.

Jurka J, Kapitonov VV, Pavlicek A, Klonowski P, Kohany O, Walichiewicz J. 2005. Repbase Update, a database of eukaryotic repetitive elements. Cytogenet. Genome Res. 110:462–467.

Kim D, Pertea G, Trapnell C, Pimentel H, Kelley R, Salzberg SL. 2013. TopHat2: accurate alignment of transcriptomes in the presence of insertions, deletions and gene fusions. Genome Biol. 14:R36.

Parker DJ, Ritchie MG, Kankare M. 2016. Preparing for Winter: the Transcriptomic Response Associated with Different Day Lengths in *Drosophila montana*. G3 GenesGenomesGenetics:g3.116.027870.

Parker DJ, Vesala L, Ritchie MG, Laiho A, Hoikkala A, Kankare M. 2015. How consistent are the transcriptome changes associated with cold acclimation in two species of the *Drosophila virilis* group? Heredity.

Sasson A, Michael TP. 2010. Filtering error from SOLiD Output. Bioinformatics 26:849–850.

Smit AFA, Hubley R, Green P. 2015. RepeatMasker Open-4.0. 2013–2015. Available from: http://www.repeatmasker.org.

Trapnell C, Williams BA, Pertea G, Mortazavi A, Kwan G, van Baren MJ, Salzberg SL, Wold BJ, Pachter L. 2010. Transcript assembly and quantification by RNA-Seq reveals unannotated transcripts and isoform switching during cell differentiation. Nat. Biotechnol. 28:511–515.
