# Supplementary material for: Inter and Intraspecific Genomic Divergence in Drosophila montana Shows Evidence for Cold Adaptation
Source: Genome Biol Evol. 2018 Jul 13;10(8):2086–101. doi: 10.1093/gbe/evy147 (PMC6107330; doi:10.1093/gbe/evy147)
Supplement: Supplementary Data [file evy147_suppl.zip › Supplemental_Tables_3to18.doc]

**Supplemental Table 3 | *dS*, *dN* and omega estimates for 251 genes in 13 *Drosophila* species that are better explained by a model assuming one value of omega for all “cold adapted” species and another value of omega for all “non cold adapted” species. Figures in brackets are standard errors**.

| **Status** | **Species** | ***dS*** | ***dN*** | **omega** |
| --- | --- | --- | --- | --- |
| Cold Tolerant | *D. montana* | 0.16 | 0.063 | 0.19 |
|  | *D. mojavensis* | 0.43 | 0.088 | 0.19 |
|  | *D. virilis* | 0.21 | 0.050 | 0.19 |
|  | *D. pseudoobscura* | 0.060 | 0.015 | 0.19 |
|  | *D. perisimils* | 0.078 | 0.018 | 0.19 |
|  | *D. melanogaster* | 0.055 | 0.010 | 0.19 |
| **Average** |  | **0.17** | **0.04** | **0.19** |
| Non Cold Tolerant | *D. grimshawi* | 0.43 | 0.048 | 0.10 |
|  | *D. willistoni* | 0.79 | 0.082 | 0.10 |
|  | *D. ananasssae* | 0.53 | 0.064 | 0.10 |
|  | *D. erecta* | 0.11 | 0.016 | 0.10 |
|  | *D. yakuba* | 0.18 | 0.027 | 0.10 |
|  | *D. simulans* | 0.039 | 0.011 | 0.10 |
|  | *D. sechellia* | 0.038 | 0.004 | 0.10 |
| **Average** |  | **0.30** | **0.036** | **0.10** |

**Supplemental Table 4 | Significantly (FDR < 0.1) enriched GO-terms for genes with higher dN/dS in cold tolerant species**

| **GO term** | **GO term description** | **FDR** | **GO type** |
| --- | --- | --- | --- |
| **GO:0031256** | leading edge membrane | 0.00424 | C |
| **GO:0032589** | neuron projection membrane | 0.00450 | C |
| **GO:0032590** | dendrite membrane | 0.00530 | C |
| **GO:0031253** | cell projection membrane | 0.00580 | C |
| **GO:0016328** | lateral plasma membrane | 0.01170 | C |
| **GO:0050793** | regulation of developmental process | 0.03240 | P |
| **GO:0010369** | chromocenter | 0.03300 | C |
| **GO:0022838** | substrate-specific channel activity | 0.03410 | M |
| **GO:0010092** | specification of animal organ identity | 0.03840 | P |
| **GO:0015267** | channel activity | 0.03890 | M |
| **GO:0042493** | response to drug | 0.04150 | P |
| **GO:0022803** | passive transmembrane transporter activity | 0.04870 | M |
| **GO:0005216** | ion channel activity | 0.04910 | M |
| **GO:0098687** | chromosomal region | 0.05310 | C |
| **GO:0004984** | olfactory receptor activity | 0.06350 | M |
| **GO:0009886** | post-embryonic animal morphogenesis | 0.08210 | P |
| **GO:0022891** | substrate-specific transmembrane transporter activity | 0.08350 | M |
| **GO:0022857** | transmembrane transporter activity | 0.09260 | M |
| **GO:0022892** | substrate-specific transporter activity | 0.09320 | M |
| **GO:0015103** | inorganic anion transmembrane transporter activity | 0.09480 | M |
| **GO:0060089** | molecular transducer activity | 0.09530 | M |
| **GO:0051239** | regulation of multicellular organismal process | 0.09780 | P |
| **GO:0008049** | male courtship behavior | 0.09860 | P |

**Supplemental Table 5 | Significantly (FDR < 0.1) enriched GO-terms for genes with higher dN/dS in non-cold tolerant species**

| **GO term** | **GO term description** | **FDR** | **GO type** |
| --- | --- | --- | --- |
| **GO:0005634** | nucleus | 0.000619 | C |
| **GO:0032991** | macromolecular complex | 0.00106 | C |
| **GO:0043234** | protein complex | 0.00114 | C |
| **GO:0043161** | proteasome-mediated ubiquitin-dependent protein catabolic process | 0.00277 | P |
| **GO:0044428** | nuclear part | 0.00433 | C |
| **GO:0035230** | cytoneme | 0.00562 | C |
| **GO:0008275** | gamma-tubulin small complex | 0.00587 | C |
| **GO:0031461** | cullin-RING ubiquitin ligase complex | 0.00955 | C |
| **GO:0010498** | proteasomal protein catabolic process | 0.0121 | P |
| **GO:0019941** | modification-dependent protein catabolic process | 0.0153 | P |
| **GO:0006511** | ubiquitin-dependent protein catabolic process | 0.0184 | P |
| **GO:0000151** | ubiquitin ligase complex | 0.0184 | C |
| **GO:0044451** | nucleoplasm part | 0.0192 | C |
| **GO:0051726** | regulation of cell cycle | 0.0207 | P |
| **GO:0044427** | chromosomal part | 0.0207 | C |
| **GO:0043632** | modification-dependent macromolecule catabolic process | 0.023 | P |
| **GO:0009948** | anterior/posterior axis specification | 0.0247 | P |
| **GO:0051603** | proteolysis involved in cellular protein catabolic process | 0.0283 | P |
| **GO:0000930** | gamma-tubulin complex | 0.0307 | C |
| **GO:0048523** | negative regulation of cellular process | 0.0348 | P |
| **GO:0044702** | single organism reproductive process | 0.038 | P |
| **GO:0009620** | response to fungus | 0.0397 | P |
| **GO:0048518** | positive regulation of biological process | 0.0403 | P |
| **GO:0022414** | reproductive process | 0.0414 | P |
| **GO:0090596** | sensory organ morphogenesis | 0.042 | P |
| **GO:0001745** | compound eye morphogenesis | 0.0422 | P |
| **GO:0022412** | cellular process involved in reproduction in multicellular organism | 0.0435 | P |
| **GO:0030163** | protein catabolic process | 0.0438 | P |
| **GO:0048592** | eye morphogenesis | 0.0444 | P |
| **GO:0009887** | animal organ morphogenesis | 0.0453 | P |
| **GO:0048869** | cellular developmental process | 0.0486 | P |
| **GO:0007298** | border follicle cell migration | 0.0488 | P |
| **GO:0051704** | multi-organism process | 0.0512 | P |
| **GO:0010628** | positive regulation of gene expression | 0.0553 | P |
| **GO:0022402** | cell cycle process | 0.0567 | P |
| **GO:0048519** | negative regulation of biological process | 0.0588 | P |
| **GO:0051252** | regulation of RNA metabolic process | 0.0599 | P |
| **GO:0007297** | ovarian follicle cell migration | 0.0626 | P |
| **GO:0005700** | polytene chromosome | 0.0682 | C |
| **GO:0008623** | CHRAC | 0.0698 | C |
| **GO:0000578** | embryonic axis specification | 0.0751 | P |
| **GO:0043015** | gamma-tubulin binding | 0.0755 | M |
| **GO:0010468** | regulation of gene expression | 0.0768 | P |
| **GO:0003676** | nucleic acid binding | 0.0892 | M |
| **GO:0008595** | anterior/posterior axis specification, embryo | 0.0912 | P |
| **GO:0006325** | chromatin organization | 0.0929 | P |
| **GO:0010604** | positive regulation of macromolecule metabolic process | 0.0937 | P |
| **GO:0030036** | actin cytoskeleton organization | 0.0952 | P |
| **GO:0005546** | phosphatidylinositol-4,5-bisphosphate binding | 0.0965 | M |
| **GO:0006928** | movement of cell or subcellular component | 0.0983 | P |

**Supplemental Table 6 | Significantly (FDR < 0.1) enriched functional clusters for genes with significantly higher dN/dS in cold tolerant species**

| **Cluster** | **Score** | **p** | **Annotation terms** |
| --- | --- | --- | --- |
| **1** | 2.74 | 0.00181 | Nucleotide-binding, ATP-binding, nucleotide phosphate-binding region:ATP |
| **2** | 2.59 | 0.00260 | olfactory receptor activity, dendrite membrane, odorant binding, Sensory transduction, Olfaction, sensory perception of smell, detection of chemical stimulus involved in sensory perception of smell, Olfactory receptor |
| **3** | 2.48 | 0.00332 | Transmembrane helix, integral component of membrane, Transmembrane, Membrane |
| **4** | 2.36 | 0.00438 | Glycoprotein, plasma membrane, glycosylation site:N-linked (GlcNAc...), signal peptide, transmembrane region, Cell membrane |
| **5** | 1.57 | 0.02716 | ovarian follicle cell development, Differentiation, Neurogenesis, cell fate specification, compound eye development, stem cell differentiation |
| **6** | 1.23 | 0.05925 | ventral cord development, ectoderm development, mesoderm development |
| **7** | 1.22 | 0.06091 | typical subtype, Leucine-rich repeat, Leucine-rich repeat |
| **8** | 1.17 | 0.06685 | Small GTPase superfamily, P-loop containing nucleoside triphosphate hydrolase, GTPase activity, Lipoprotein, GTP binding, Methylation, Prenylation, GDP binding, small GTPase mediated signal transduction, Small GTP-binding protein domain, Ras type |
| **9** | 1.11 | 0.07838 | mRNA binding, intracellular ribonucleoprotein complex, nucleotide binding, nucleic acid binding, RNA recognition motif domain, alpha-beta plait |
| **10** | 1.07 | 0.08500 | Cytoskeleton, Microtubule |
| **11** | 1.07 | 0.08525 | Calcium transport, calcium ion transport, Ion transport, Ion channel, Transport |

**Supplemental Table 7 | Significantly (FDR < 0.1) enriched functional clusters for genes with significantly higher dN/dS in non-cold tolerant species**

| **Cluster** | **Enrichment** | **P** | **Annotation terms** |
| --- | --- | --- | --- |
| 1 | 1.54 | 0.02915 | Disulfide bond, conserved site, EGF-like calcium-binding, Epidermal growth factor-like domain, calcium ion binding, EGF-like domain |
| 2 | 1.35 | 0.04478 | Transmembrane, Disulfide bond, glycosylation site:N-linked (GlcNAc...), apical plasma membrane, protein heterodimerization activity, axon guidance, signal peptide, heterophilic cell-cell adhesion via plasma membrane cell adhesion molecules, border follicle cell migration, receptor activity, Glycoprotein, integral component of membrane, cell adhesion, Transmembrane helix |
| 3 | 1.13 | 0.07377 | microtubule associated complex, Cytoplasm, Cytoskeleton, mitotic spindle organization, Microtubule |

**Supplemental Table 8| Significantly (FDR < 0.1) enriched GO-terms for genes showing high dN/dS between *D. montana* and *D. virilis***

| **GO term** | **GO term description** | **FDR** | **GO type** |
| --- | --- | --- | --- |
| **GO:0005576** | extracellular region | 3.24E-11 | C |
| **GO:0008061** | chitin binding | 2.58E-08 | M |
| **GO:0005549** | odorant binding | 5.53E-08 | M |
| **GO:0007606** | sensory perception of chemical stimulus | 2.04E-07 | P |
| **GO:0032504** | multicellular organism reproduction | 7.37E-07 | P |
| **GO:1901071** | glucosamine-containing compound metabolic process | 9.82E-07 | P |
| **GO:0006030** | chitin metabolic process | 1.22E-06 | P |
| **GO:0006040** | amino sugar metabolic process | 1.28E-06 | P |
| **GO:0000003** | reproduction | 1.65E-06 | P |
| **GO:0009593** | detection of chemical stimulus | 2.55E-06 | P |
| **GO:0005615** | extracellular space | 7.00E-06 | C |
| **GO:0006022** | aminoglycan metabolic process | 0.0002 | P |
| **GO:0044421** | extracellular region part | 0.0003 | C |
| **GO:0004984** | olfactory receptor activity | 0.0004 | M |
| **GO:0032590** | dendrite membrane | 0.0004 | C |
| **GO:0032589** | neuron projection membrane | 0.0012 | C |
| **GO:0031256** | leading edge membrane | 0.0022 | C |
| **GO:0042600** | chorion | 0.0038 | C |
| **GO:0030312** | external encapsulating structure | 0.0038 | C |
| **GO:0031253** | cell projection membrane | 0.0123 | C |
| **GO:0008527** | taste receptor activity | 0.0165 | M |
| **GO:0050909** | sensory perception of taste | 0.0203 | P |
| **GO:0050907** | detection of chemical stimulus involved in sensory perception | 0.0648 | P |

**Supplemental Table 9 | Significantly (FDR < 0.1) enriched functional clusters for genes with the top 10% highest dN/dS values between *D. montana* and *D. virilis***

| **Cluster** | **Enrichment** | | **p** | **Annotation terms** |
| --- | --- | --- | --- | --- |
| **1** | 4.40 | 4.01E-05 | | chitin binding, chitin metabolic process |
| **2** | 2.72 | 0.00192 | | odorant binding, Pheromone/general odorant binding protein, sensory perception of chemical stimulus |
| **3** | 2.17 | 0.00669 | | Olfactory receptor, transmembrane region, dendrite membrane, Transducer, Olfaction, Receptor, Sensory transduction, sensory perception of smell, olfactory receptor activity, plasma membrane, detection of chemical stimulus involved in sensory perception of smell, odorant binding |
| **4** | 1.79 | 0.01627 | | carbohydrate binding, C-type lectin fold, C-type lectin |
| **5** | 1.50 | 0.03134 | | chitin-based cuticle development, structural constituent of cuticle, extracellular matrix, structural constituent of chitin-based larval cuticle, Insect cuticle protein |
| **6** | 1.44 | 0.03603 | | Helix-loop-helix motif, Myc-type, protein dimerization activity, basic helix-loop-helix (bHLH) domain |
| **7** | 1.42 | 0.03825 | | Fibrinogen, alpha/beta/gamma chain, C-terminal globular domain |
| **8** | 1.03 | 0.09262 | | CAP domain, SM00198:SCP, Allergen V5/Tpx-1-related |
| **9** | 1.01 | 0.09816 | | sequence-specific DNA binding, transcription factor activity, DNA-binding |

**Supplemental Table 10 | Significantly (FDR < 0.1) enriched GO-terms for genes showing low dN/dS between *D. montana* and *D. virilis***

| **GO term** | **GO term description** | | **FDR** | **GO type** |
| --- | --- | --- | --- | --- |
| GO:0032991 | macromolecular complex | 2.99E-66 | | C |
| GO:0044464 | cell part | 4.00E-54 | | C |
| GO:0044424 | intracellular part | 7.74E-45 | | C |
| GO:0009987 | cellular process | 1.43E-43 | | P |
| GO:0043234 | protein complex | 1.35E-39 | | C |
| GO:0044446 | intracellular organelle part | 2.14E-35 | | C |
| GO:0044422 | organelle part | 1.21E-34 | | C |
| GO:0065007 | biological regulation | 8.08E-29 | | P |
| GO:0044763 | single-organism cellular process | 1.61E-28 | | P |
| GO:0002181 | cytoplasmic translation | 4.95E-28 | | P |
| GO:0048869 | cellular developmental process | 5.60E-28 | | P |
| GO:0044767 | single-organism developmental process | 2.48E-25 | | P |
| GO:0032502 | developmental process | 6.80E-25 | | P |
| GO:0044699 | single-organism process | 1.81E-24 | | P |
| GO:0050789 | regulation of biological process | 3.41E-24 | | P |
| GO:0005840 | ribosome | 6.61E-23 | | C |
| GO:0044445 | cytosolic part | 1.15E-22 | | C |
| GO:0044237 | cellular metabolic process | 2.21E-22 | | P |
| GO:0044260 | cellular macromolecule metabolic process | 1.11E-21 | | P |
| GO:0050794 | regulation of cellular process | 1.62E-20 | | P |
| GO:0030529 | intracellular ribonucleoprotein complex | 1.90E-20 | | C |
| GO:1990904 | ribonucleoprotein complex | 2.11E-20 | | C |
| GO:0044428 | nuclear part | 2.26E-20 | | C |
| GO:0000166 | nucleotide binding | 2.77E-20 | | M |
| GO:0043168 | anion binding | 2.85E-20 | | M |
| GO:0035639 | purine ribonucleoside triphosphate binding | 3.03E-20 | | M |
| GO:1901265 | nucleoside phosphate binding | 3.23E-20 | | M |
| GO:0032555 | purine ribonucleotide binding | 3.58E-20 | | M |
| GO:0017076 | purine nucleotide binding | 4.77E-20 | | M |
| GO:0032553 | ribonucleotide binding | 6.64E-20 | | M |
| GO:0036094 | small molecule binding | 6.88E-19 | | M |
| GO:1902494 | catalytic complex | 7.11E-18 | | C |
| GO:0032549 | ribonucleoside binding | 7.82E-18 | | M |
| GO:0044444 | cytoplasmic part | 8.84E-18 | | C |
| GO:0006810 | transport | 1.09E-17 | | P |
| GO:0005525 | GTP binding | 6.59E-17 | | M |
| GO:0032550 | purine ribonucleoside binding | 7.25E-17 | | M |
| GO:0019001 | guanyl nucleotide binding | 8.09E-17 | | M |
| GO:0016192 | vesicle-mediated transport | 1.07E-16 | | P |
| GO:0001882 | nucleoside binding | 1.29E-16 | | M |
| GO:0032561 | guanyl ribonucleotide binding | 1.49E-16 | | M |
| GO:0051234 | establishment of localization | 1.77E-16 | | P |
| GO:0051179 | localization | 1.78E-16 | | P |
| GO:0071840 | cellular component organization or biogenesis | 1.96E-16 | | P |
| GO:0044391 | ribosomal subunit | 2.18E-16 | | C |
| GO:0016043 | cellular component organization | 3.66E-16 | | P |
| GO:0001883 | purine nucleoside binding | 3.91E-16 | | M |
| GO:0022626 | cytosolic ribosome | 4.69E-16 | | C |
| GO:0006412 | translation | 5.48E-16 | | P |
| GO:0003735 | structural constituent of ribosome | 5.72E-16 | | M |
| GO:0043043 | peptide biosynthetic process | 1.21E-15 | | P |
| GO:0005515 | protein binding | 1.67E-15 | | M |
| GO:0022627 | cytosolic small ribosomal subunit | 6.49E-15 | | C |
| GO:0043604 | amide biosynthetic process | 1.27E-14 | | P |
| GO:0003924 | GTPase activity | 1.57E-14 | | M |
| GO:0044451 | nucleoplasm part | 4.46E-14 | | C |
| GO:0071011 | precatalytic spliceosome | 5.32E-14 | | C |
| GO:0009059 | macromolecule biosynthetic process | 6.13E-14 | | P |
| GO:0005875 | microtubule associated complex | 1.42E-13 | | C |
| GO:0006518 | peptide metabolic process | 1.90E-13 | | P |
| GO:0005681 | spliceosomal complex | 2.00E-13 | | C |
| GO:0017111 | nucleoside-triphosphatase activity | 2.27E-13 | | M |
| GO:0007264 | small GTPase mediated signal transduction | 3.18E-13 | | P |
| GO:0005737 | cytoplasm | 5.30E-13 | | C |
| GO:0005886 | plasma membrane | 5.34E-13 | | C |
| GO:0016462 | pyrophosphatase activity | 7.45E-13 | | M |
| GO:0044430 | cytoskeletal part | 8.45E-13 | | C |
| GO:0022625 | cytosolic large ribosomal subunit | 8.51E-13 | | C |
| GO:0097367 | carbohydrate derivative binding | 1.45E-12 | | M |
| GO:0016818 | hydrolase activity, acting on acid anhydrides, in phosphorus-containing anhydrides | 2.78E-12 | | M |
| GO:0008380 | RNA splicing | 2.88E-12 | | P |
| GO:0034645 | cellular macromolecule biosynthetic process | 2.91E-12 | | P |
| GO:0006397 | mRNA processing | 3.43E-12 | | P |
| GO:0016071 | mRNA metabolic process | 5.11E-12 | | P |
| GO:0016817 | hydrolase activity, acting on acid anhydrides | 5.61E-12 | | M |
| GO:0051649 | establishment of localization in cell | 5.91E-12 | | P |
| GO:0043603 | cellular amide metabolic process | 6.43E-12 | | P |
| GO:0043170 | macromolecule metabolic process | 6.79E-12 | | P |
| GO:0044267 | cellular protein metabolic process | 6.87E-12 | | P |
| GO:0030154 | cell differentiation | 6.94E-12 | | P |
| GO:0000398 | mRNA splicing, via spliceosome | 7.25E-12 | | P |
| GO:0000377 | RNA splicing, via transesterification reactions with bulged adenosine as nucleophile | 7.46E-12 | | P |
| GO:0000375 | RNA splicing, via transesterification reactions | 8.50E-12 | | P |
| GO:0044271 | cellular nitrogen compound biosynthetic process | 1.20E-11 | | P |
| GO:0022412 | cellular process involved in reproduction in multicellular organism | 1.26E-11 | | P |
| GO:0044238 | primary metabolic process | 1.27E-11 | | P |
| GO:0022402 | cell cycle process | 1.44E-11 | | P |
| GO:0044702 | single organism reproductive process | 1.61E-11 | | P |
| GO:0005198 | structural molecule activity | 1.77E-11 | | M |
| GO:0022414 | reproductive process | 1.94E-11 | | P |
| GO:0043226 | organelle | 1.96E-11 | | C |
| GO:0043229 | intracellular organelle | 1.97E-11 | | C |
| GO:0035556 | intracellular signal transduction | 2.20E-11 | | P |
| GO:0022008 | neurogenesis | 2.32E-11 | | P |
| GO:0005488 | binding | 2.54E-11 | | M |
| GO:0048522 | positive regulation of cellular process | 2.60E-11 | | P |
| GO:0043232 | intracellular non-membrane-bounded organelle | 2.68E-11 | | C |
| GO:0043228 | non-membrane-bounded organelle | 2.77E-11 | | C |
| GO:0016070 | RNA metabolic process | 3.05E-11 | | P |
| GO:0065008 | regulation of biological quality | 3.07E-11 | | P |
| GO:0048518 | positive regulation of biological process | 3.83E-11 | | P |
| GO:0048856 | anatomical structure development | 4.30E-11 | | P |
| GO:0034641 | cellular nitrogen compound metabolic process | 4.93E-11 | | P |
| GO:0044765 | single-organism transport | 5.00E-11 | | P |
| GO:0051128 | regulation of cellular component organization | 5.01E-11 | | P |
| GO:1902578 | single-organism localization | 5.26E-11 | | P |
| GO:0019222 | regulation of metabolic process | 6.40E-11 | | P |
| GO:0009653 | anatomical structure morphogenesis | 6.98E-11 | | P |
| GO:0005575 | cellular_component | 1.76E-10 | | C |
| GO:0046907 | intracellular transport | 1.82E-10 | | P |
| GO:0032559 | adenyl ribonucleotide binding | 1.92E-10 | | M |
| GO:0030554 | adenyl nucleotide binding | 2.35E-10 | | M |
| GO:0005524 | ATP binding | 2.51E-10 | | M |
| GO:0071013 | catalytic step 2 spliceosome | 2.64E-10 | | C |
| GO:1903047 | mitotic cell cycle process | 3.94E-10 | | P |
| GO:0031323 | regulation of cellular metabolic process | 4.42E-10 | | P |
| GO:0050793 | regulation of developmental process | 5.65E-10 | | P |
| GO:0080090 | regulation of primary metabolic process | 6.38E-10 | | P |
| GO:0051641 | cellular localization | 6.45E-10 | | P |
| GO:0007165 | signal transduction | 6.98E-10 | | P |
| GO:0044707 | single-multicellular organism process | 7.48E-10 | | P |
| GO:0003006 | developmental process involved in reproduction | 8.55E-10 | | P |
| GO:0071704 | organic substance metabolic process | 1.14E-09 | | P |
| GO:0007010 | cytoskeleton organization | 1.19E-09 | | P |
| GO:0019538 | protein metabolic process | 1.20E-09 | | P |
| GO:0006996 | organelle organization | 1.21E-09 | | P |
| GO:1901576 | organic substance biosynthetic process | 1.21E-09 | | P |
| GO:0097159 | organic cyclic compound binding | 1.22E-09 | | M |
| GO:1901363 | heterocyclic compound binding | 1.31E-09 | | M |
| GO:0023051 | regulation of signaling | 1.42E-09 | | P |
| GO:0010646 | regulation of cell communication | 1.44E-09 | | P |
| GO:0048523 | negative regulation of cellular process | 1.45E-09 | | P |
| GO:0048519 | negative regulation of biological process | 2.04E-09 | | P |
| GO:0003723 | RNA binding | 2.05E-09 | | M |
| GO:0007265 | Ras protein signal transduction | 2.40E-09 | | P |
| GO:0015935 | small ribosomal subunit | 2.56E-09 | | C |
| GO:0009058 | biosynthetic process | 2.76E-09 | | P |
| GO:0006367 | transcription initiation from RNA polymerase II promoter | 3.10E-09 | | P |
| GO:0048583 | regulation of response to stimulus | 3.13E-09 | | P |
| GO:0032989 | cellular component morphogenesis | 4.28E-09 | | P |
| GO:1901566 | organonitrogen compound biosynthetic process | 5.41E-09 | | P |
| GO:0008152 | metabolic process | 6.81E-09 | | P |
| GO:0098796 | membrane protein complex | 7.68E-09 | | C |
| GO:0044249 | cellular biosynthetic process | 7.86E-09 | | P |
| GO:0005200 | structural constituent of cytoskeleton | 8.50E-09 | | M |
| GO:0060255 | regulation of macromolecule metabolic process | 1.26E-08 | | P |
| GO:0009966 | regulation of signal transduction | 1.26E-08 | | P |
| GO:0070727 | cellular macromolecule localization | 1.30E-08 | | P |
| GO:0006366 | transcription from RNA polymerase II promoter | 1.38E-08 | | P |
| GO:0048468 | cell development | 1.64E-08 | | P |
| GO:0033036 | macromolecule localization | 1.70E-08 | | P |
| GO:0006897 | endocytosis | 1.90E-08 | | P |
| GO:0010604 | positive regulation of macromolecule metabolic process | 3.10E-08 | | P |
| GO:0051239 | regulation of multicellular organismal process | 3.85E-08 | | P |
| GO:0036211 | protein modification process | 3.89E-08 | | P |
| GO:0006464 | cellular protein modification process | 3.93E-08 | | P |
| GO:0040011 | locomotion | 5.35E-08 | | P |
| GO:0006352 | DNA-templated transcription, initiation | 5.55E-08 | | P |
| GO:0051960 | regulation of nervous system development | 6.95E-08 | | P |
| GO:1901987 | regulation of cell cycle phase transition | 9.35E-08 | | P |
| GO:1901990 | regulation of mitotic cell cycle phase transition | 9.45E-08 | | P |
| GO:0006396 | RNA processing | 9.52E-08 | | P |
| GO:0032774 | RNA biosynthetic process | 1.10E-07 | | P |
| GO:0006928 | movement of cell or subcellular component | 1.30E-07 | | P |
| GO:0031572 | G2 DNA damage checkpoint | 1.36E-07 | | P |
| GO:0007095 | mitotic G2 DNA damage checkpoint | 1.37E-07 | | P |
| GO:0006807 | nitrogen compound metabolic process | 1.54E-07 | | P |
| GO:0009893 | positive regulation of metabolic process | 1.55E-07 | | P |
| GO:2000026 | regulation of multicellular organismal development | 1.64E-07 | | P |
| GO:0048513 | animal organ development | 1.82E-07 | | P |
| GO:0043227 | membrane-bounded organelle | 1.92E-07 | | C |
| GO:0016020 | membrane | 1.96E-07 | | C |
| GO:0010972 | negative regulation of G2/M transition of mitotic cell cycle | 2.25E-07 | | P |
| GO:1902750 | negative regulation of cell cycle G2/M phase transition | 2.27E-07 | | P |
| GO:0044818 | mitotic G2/M transition checkpoint | 2.29E-07 | | P |
| GO:0007015 | actin filament organization | 2.45E-07 | | P |
| GO:0031325 | positive regulation of cellular metabolic process | 2.47E-07 | | P |
| GO:1901360 | organic cyclic compound metabolic process | 2.59E-07 | | P |
| GO:0007297 | ovarian follicle cell migration | 2.72E-07 | | P |
| GO:0097435 | supramolecular fiber organization | 3.11E-07 | | P |
| GO:0007281 | germ cell development | 3.36E-07 | | P |
| GO:0051246 | regulation of protein metabolic process | 3.53E-07 | | P |
| GO:0032990 | cell part morphogenesis | 3.60E-07 | | P |
| GO:0030532 | small nuclear ribonucleoprotein complex | 4.09E-07 | | C |
| GO:0043412 | macromolecule modification | 4.22E-07 | | P |
| GO:0006725 | cellular aromatic compound metabolic process | 4.51E-07 | | P |
| GO:1902749 | regulation of cell cycle G2/M phase transition | 5.03E-07 | | P |
| GO:0010389 | regulation of G2/M transition of mitotic cell cycle | 5.07E-07 | | P |
| GO:0051716 | cellular response to stimulus | 5.23E-07 | | P |
| GO:0048812 | neuron projection morphogenesis | 5.24E-07 | | P |
| GO:0048858 | cell projection morphogenesis | 5.29E-07 | | P |
| GO:0044773 | mitotic DNA damage checkpoint | 5.31E-07 | | P |
| GO:0006139 | nucleobase-containing compound metabolic process | 8.06E-07 | | P |
| GO:0044774 | mitotic DNA integrity checkpoint | 8.79E-07 | | P |
| GO:0030036 | actin cytoskeleton organization | 9.38E-07 | | P |
| GO:1902580 | single-organism cellular localization | 0.00000112 | | P |
| GO:0071705 | nitrogen compound transport | 0.00000117 | | P |
| GO:0008104 | protein localization | 0.00000124 | | P |
| GO:0007298 | border follicle cell migration | 0.00000129 | | P |
| GO:0015934 | large ribosomal subunit | 0.00000142 | | C |
| GO:0090304 | nucleic acid metabolic process | 0.00000151 | | P |
| GO:0008540 | proteasome regulatory particle, base subcomplex | 0.00000154 | | C |
| GO:0005634 | nucleus | 0.00000154 | | C |
| GO:0002064 | epithelial cell development | 0.00000172 | | P |
| GO:0030030 | cell projection organization | 0.00000172 | | P |
| GO:0034654 | nucleobase-containing compound biosynthetic process | 0.00000178 | | P |
| GO:1901988 | negative regulation of cell cycle phase transition | 0.00000205 | | P |
| GO:1901991 | negative regulation of mitotic cell cycle phase transition | 0.00000207 | | P |
| GO:0006909 | phagocytosis | 0.00000246 | | P |
| GO:0032879 | regulation of localization | 0.00000246 | | P |
| GO:0030029 | actin filament-based process | 0.00000248 | | P |
| GO:0000077 | DNA damage checkpoint | 0.00000253 | | P |
| GO:1902589 | single-organism organelle organization | 0.00000256 | | P |
| GO:0007154 | cell communication | 0.00000266 | | P |
| GO:0044087 | regulation of cellular component biogenesis | 0.00000273 | | P |
| GO:0006886 | intracellular protein transport | 0.00000284 | | P |
| GO:0033554 | cellular response to stress | 0.00000301 | | P |
| GO:0006950 | response to stress | 0.00000307 | | P |
| GO:0016477 | cell migration | 0.00000308 | | P |
| GO:0031329 | regulation of cellular catabolic process | 0.00000392 | | P |
| GO:0002066 | columnar/cuboidal epithelial cell development | 0.00000399 | | P |
| GO:0046483 | heterocycle metabolic process | 0.00000412 | | P |
| GO:0010564 | regulation of cell cycle process | 0.00000471 | | P |
| GO:0007163 | establishment or maintenance of cell polarity | 0.00000482 | | P |
| GO:0019438 | aromatic compound biosynthetic process | 0.00000487 | | P |
| GO:1990234 | transferase complex | 0.00000517 | | C |
| GO:0007093 | mitotic cell cycle checkpoint | 0.00000525 | | P |
| GO:0032268 | regulation of cellular protein metabolic process | 0.00000543 | | P |
| GO:0001667 | ameboidal-type cell migration | 0.00000553 | | P |
| GO:0016592 | mediator complex | 0.00000597 | | C |
| GO:0045595 | regulation of cell differentiation | 0.00000599 | | P |
| GO:0010631 | epithelial cell migration | 0.00000628 | | P |
| GO:0030865 | cortical cytoskeleton organization | 0.0000066 | | P |
| GO:0051130 | positive regulation of cellular component organization | 0.0000066 | | P |
| GO:0051247 | positive regulation of protein metabolic process | 0.00000673 | | P |
| GO:0070647 | protein modification by small protein conjugation or removal | 0.00000679 | | P |
| GO:0048870 | cell motility | 0.00000684 | | P |
| GO:0030707 | ovarian follicle cell development | 0.00000748 | | P |
| GO:0031570 | DNA integrity checkpoint | 0.00000766 | | P |
| GO:0040008 | regulation of growth | 0.0000095 | | P |
| GO:0045930 | negative regulation of mitotic cell cycle | 0.0000101 | | P |
| GO:0051726 | regulation of cell cycle | 0.0000103 | | P |
| GO:0009894 | regulation of catabolic process | 0.0000138 | | P |
| GO:0090575 | RNA polymerase II transcription factor complex | 0.0000138 | | C |
| GO:0010948 | negative regulation of cell cycle process | 0.0000141 | | P |
| GO:0043167 | ion binding | 0.0000154 | | M |
| GO:0023056 | positive regulation of signaling | 0.0000175 | | P |
| GO:0010647 | positive regulation of cell communication | 0.0000176 | | P |
| GO:0006974 | cellular response to DNA damage stimulus | 0.0000179 | | P |
| GO:0000075 | cell cycle checkpoint | 0.0000182 | | P |
| GO:0050767 | regulation of neurogenesis | 0.0000186 | | P |
| GO:0097659 | nucleic acid-templated transcription | 0.0000194 | | P |
| GO:0061640 | cytoskeleton-dependent cytokinesis | 0.0000194 | | P |
| GO:0006351 | transcription, DNA-templated | 0.0000195 | | P |
| GO:0000910 | cytokinesis | 0.0000195 | | P |
| GO:0072659 | protein localization to plasma membrane | 0.0000217 | | P |
| GO:0032501 | multicellular organismal process | 0.0000218 | | P |
| GO:0032270 | positive regulation of cellular protein metabolic process | 0.0000228 | | P |
| GO:0022610 | biological adhesion | 0.0000243 | | P |
| GO:0044425 | membrane part | 0.0000243 | | C |
| GO:0032456 | endocytic recycling | 0.0000267 | | P |
| GO:0018130 | heterocycle biosynthetic process | 0.000027 | | P |
| GO:0030866 | cortical actin cytoskeleton organization | 0.0000283 | | P |
| GO:0030054 | cell junction | 0.0000283 | | C |
| GO:1902582 | single-organism intracellular transport | 0.0000291 | | P |
| GO:0007346 | regulation of mitotic cell cycle | 0.0000316 | | P |
| GO:0031399 | regulation of protein modification process | 0.0000317 | | P |
| GO:0015031 | protein transport | 0.0000317 | | P |
| GO:0045184 | establishment of protein localization | 0.000032 | | P |
| GO:0005667 | transcription factor complex | 0.0000322 | | C |
| GO:0048638 | regulation of developmental growth | 0.0000338 | | P |
| GO:0043231 | intracellular membrane-bounded organelle | 0.0000371 | | C |
| GO:0005829 | cytosol | 0.0000372 | | C |
| GO:0000902 | cell morphogenesis | 0.0000393 | | P |
| GO:0000281 | mitotic cytokinesis | 0.0000427 | | P |
| GO:0031982 | vesicle | 0.0000427 | | C |
| GO:0015833 | peptide transport | 0.000043 | | P |
| GO:1903311 | regulation of mRNA metabolic process | 0.0000441 | | P |
| GO:0034613 | cellular protein localization | 0.0000448 | | P |
| GO:0042886 | amide transport | 0.000045 | | P |
| GO:0051298 | centrosome duplication | 0.0000478 | | P |
| GO:0042995 | cell projection | 0.0000489 | | C |
| GO:0050684 | regulation of mRNA processing | 0.0000494 | | P |
| GO:0005694 | chromosome | 0.0000495 | | C |
| GO:0048584 | positive regulation of response to stimulus | 0.0000499 | | P |
| GO:0001104 | RNA polymerase II transcription cofactor activity | 0.0000505 | | M |
| GO:0071702 | organic substance transport | 0.0000514 | | P |
| GO:0005838 | proteasome regulatory particle | 0.0000529 | | C |
| GO:0060284 | regulation of cell development | 0.0000543 | | P |
| GO:0048024 | regulation of mRNA splicing, via spliceosome | 0.0000554 | | P |
| GO:0007155 | cell adhesion | 0.000056 | | P |
| GO:0120025 | plasma membrane bounded cell projection | 0.0000651 | | C |
| GO:0051603 | proteolysis involved in cellular protein catabolic process | 0.0000714 | | P |
| GO:0048646 | anatomical structure formation involved in morphogenesis | 0.0000781 | | P |
| GO:0001076 | transcription factor activity, RNA polymerase II transcription factor binding | 0.0000805 | | M |
| GO:0050807 | regulation of synapse organization | 0.0000806 | | P |
| GO:0000988 | transcription factor activity, protein binding | 0.0000825 | | M |
| GO:0009886 | post-embryonic animal morphogenesis | 0.0000833 | | P |
| GO:0044248 | cellular catabolic process | 0.0000835 | | P |
| GO:0040007 | growth | 0.0000838 | | P |
| GO:0031410 | cytoplasmic vesicle | 0.0000859 | | C |
| GO:1901362 | organic cyclic compound biosynthetic process | 0.0000867 | | P |
| GO:0097708 | intracellular vesicle | 0.0000875 | | C |
| GO:0051094 | positive regulation of developmental process | 0.0000912 | | P |
| GO:0050896 | response to stimulus | 0.0000935 | | P |
| GO:0001763 | morphogenesis of a branching structure | 0.0000936 | | P |
| GO:0048140 | male germ-line cyst encapsulation | 0.000094 | | P |
| GO:0010883 | regulation of lipid storage | 0.0000955 | | P |
| GO:0005912 | adherens junction | 0.0000993 | | C |
| GO:0051962 | positive regulation of nervous system development | 0.000101 | | P |
| GO:0070161 | anchoring junction | 0.000101 | | C |
| GO:0044265 | cellular macromolecule catabolic process | 0.000103 | | P |
| GO:0045786 | negative regulation of cell cycle | 0.000115 | | P |
| GO:0035114 | imaginal disc-derived appendage morphogenesis | 0.000128 | | P |
| GO:0005811 | lipid particle | 0.000128 | | C |
| GO:0000228 | nuclear chromosome | 0.000133 | | C |
| GO:0019941 | modification-dependent protein catabolic process | 0.000137 | | P |
| GO:0022603 | regulation of anatomical structure morphogenesis | 0.000138 | | P |
| GO:0006511 | ubiquitin-dependent protein catabolic process | 0.000138 | | P |
| GO:0043632 | modification-dependent macromolecule catabolic process | 0.000139 | | P |
| GO:0055067 | monovalent inorganic cation homeostasis | 0.000142 | | P |
| GO:0006885 | regulation of pH | 0.000143 | | P |
| GO:0044448 | cell cortex part | 0.000151 | | C |
| GO:0016331 | morphogenesis of embryonic epithelium | 0.000152 | | P |
| GO:0007391 | dorsal closure | 0.000152 | | P |
| GO:0007276 | gamete generation | 0.000153 | | P |
| GO:0008565 | protein transporter activity | 0.000156 | | M |
| GO:0097525 | spliceosomal snRNP complex | 0.000156 | | C |
| GO:0031324 | negative regulation of cellular metabolic process | 0.000162 | | P |
| GO:0061138 | morphogenesis of a branching epithelium | 0.000167 | | P |
| GO:0048754 | branching morphogenesis of an epithelial tube | 0.000167 | | P |
| GO:0051050 | positive regulation of transport | 0.000171 | | P |
| GO:0031344 | regulation of cell projection organization | 0.000193 | | P |
| GO:0000381 | regulation of alternative mRNA splicing, via spliceosome | 0.000195 | | P |
| GO:0003824 | catalytic activity | 0.000204 | | M |
| GO:0009892 | negative regulation of metabolic process | 0.00021 | | P |
| GO:0048729 | tissue morphogenesis | 0.000215 | | P |
| GO:0015197 | peptide transporter activity | 0.000219 | | M |
| GO:0007476 | imaginal disc-derived wing morphogenesis | 0.000222 | | P |
| GO:0043484 | regulation of RNA splicing | 0.000225 | | P |
| GO:0009968 | negative regulation of signal transduction | 0.000225 | | P |
| GO:0022607 | cellular component assembly | 0.000231 | | P |
| GO:0006325 | chromatin organization | 0.000239 | | P |
| GO:0007267 | cell-cell signaling | 0.000244 | | P |
| GO:0043161 | proteasome-mediated ubiquitin-dependent protein catabolic process | 0.000245 | | P |
| GO:0035120 | post-embryonic appendage morphogenesis | 0.000256 | | P |
| GO:0051049 | regulation of transport | 0.000261 | | P |
| GO:0016324 | apical plasma membrane | 0.000266 | | C |
| GO:0009887 | animal organ morphogenesis | 0.000271 | | P |
| GO:0045937 | positive regulation of phosphate metabolic process | 0.000274 | | P |
| GO:0010562 | positive regulation of phosphorus metabolic process | 0.000275 | | P |
| GO:0004674 | protein serine/threonine kinase activity | 0.000278 | | M |
| GO:1990778 | protein localization to cell periphery | 0.00028 | | P |
| GO:0023061 | signal release | 0.000288 | | P |
| GO:0010468 | regulation of gene expression | 0.000298 | | P |
| GO:0001934 | positive regulation of protein phosphorylation | 0.000299 | | P |
| GO:0003729 | mRNA binding | 0.000303 | | M |
| GO:0009967 | positive regulation of signal transduction | 0.000311 | | P |
| GO:0035107 | appendage morphogenesis | 0.000312 | | P |
| GO:0007292 | female gamete generation | 0.000317 | | P |
| GO:0060446 | branching involved in open tracheal system development | 0.000318 | | P |
| GO:0048477 | oogenesis | 0.000318 | | P |
| GO:0005938 | cell cortex | 0.000318 | | C |
| GO:0099568 | cytoplasmic region | 0.000323 | | C |
| GO:0044700 | single organism signaling | 0.000341 | | P |
| GO:0044802 | single-organism membrane organization | 0.000353 | | P |
| GO:0001932 | regulation of protein phosphorylation | 0.000358 | | P |
| GO:0002009 | morphogenesis of an epithelium | 0.00037 | | P |
| GO:0045927 | positive regulation of growth | 0.000373 | | P |
| GO:1902531 | regulation of intracellular signal transduction | 0.000375 | | P |
| GO:0042127 | regulation of cell proliferation | 0.000376 | | P |
| GO:0034332 | adherens junction organization | 0.000381 | | P |
| GO:0048138 | germ-line cyst encapsulation | 0.000389 | | P |
| GO:0051489 | regulation of filopodium assembly | 0.00039 | | P |
| GO:0050657 | nucleic acid transport | 0.000391 | | P |
| GO:0010498 | proteasomal protein catabolic process | 0.000392 | | P |
| GO:0050658 | RNA transport | 0.000392 | | P |
| GO:0022604 | regulation of cell morphogenesis | 0.000413 | | P |
| GO:0051452 | intracellular pH reduction | 0.000413 | | P |
| GO:0051453 | regulation of intracellular pH | 0.000414 | | P |
| GO:0051171 | regulation of nitrogen compound metabolic process | 0.000414 | | P |
| GO:0007389 | pattern specification process | 0.000414 | | P |
| GO:0045851 | pH reduction | 0.000416 | | P |
| GO:0061024 | membrane organization | 0.000416 | | P |
| GO:0016887 | ATPase activity | 0.000416 | | M |
| GO:0016310 | phosphorylation | 0.000417 | | P |
| GO:0001505 | regulation of neurotransmitter levels | 0.000427 | | P |
| GO:0030163 | protein catabolic process | 0.000428 | | P |
| GO:0060429 | epithelium development | 0.000429 | | P |
| GO:0099531 | presynaptic process involved in chemical synaptic transmission | 0.000429 | | P |
| GO:0043933 | macromolecular complex subunit organization | 0.00043 | | P |
| GO:0044297 | cell body | 0.00043 | | C |
| GO:0044433 | cytoplasmic vesicle part | 0.00044 | | C |
| GO:1903036 | positive regulation of response to wounding | 0.000464 | | P |
| GO:0023052 | signaling | 0.000465 | | P |
| GO:0048585 | negative regulation of response to stimulus | 0.000487 | | P |
| GO:1901564 | organonitrogen compound metabolic process | 0.000491 | | P |
| GO:0043025 | neuronal cell body | 0.000499 | | C |
| GO:0007409 | axonogenesis | 0.000517 | | P |
| GO:0051236 | establishment of RNA localization | 0.000519 | | P |
| GO:0008593 | regulation of Notch signaling pathway | 0.000537 | | P |
| GO:0042327 | positive regulation of phosphorylation | 0.000554 | | P |
| GO:0009057 | macromolecule catabolic process | 0.000558 | | P |
| GO:0051297 | centrosome organization | 0.000558 | | P |
| GO:0019219 | regulation of nucleobase-containing compound metabolic process | 0.000559 | | P |
| GO:0033043 | regulation of organelle organization | 0.000563 | | P |
| GO:1903034 | regulation of response to wounding | 0.00057 | | P |
| GO:0032940 | secretion by cell | 0.000574 | | P |
| GO:0006357 | regulation of transcription from RNA polymerase II promoter | 0.000575 | | P |
| GO:0007017 | microtubule-based process | 0.000601 | | P |
| GO:0051252 | regulation of RNA metabolic process | 0.000606 | | P |
| GO:0099537 | trans-synaptic signaling | 0.000615 | | P |
| GO:0007293 | germarium-derived egg chamber formation | 0.000616 | | P |
| GO:0099536 | synaptic signaling | 0.000617 | | P |
| GO:0042325 | regulation of phosphorylation | 0.000625 | | P |
| GO:0044798 | nuclear transcription factor complex | 0.000641 | | C |
| GO:0099643 | signal release from synapse | 0.000679 | | P |
| GO:0030705 | cytoskeleton-dependent intracellular transport | 0.000695 | | P |
| GO:0006468 | protein phosphorylation | 0.000696 | | P |
| GO:0060560 | developmental growth involved in morphogenesis | 0.000753 | | P |
| GO:0045216 | cell-cell junction organization | 0.000777 | | P |
| GO:0034330 | cell junction organization | 0.00078 | | P |
| GO:0043408 | regulation of MAPK cascade | 0.000798 | | P |
| GO:0000989 | transcription factor activity, transcription factor binding | 0.000803 | | M |
| GO:0031330 | negative regulation of cellular catabolic process | 0.000815 | | P |
| GO:0008135 | translation factor activity, RNA binding | 0.000817 | | M |
| GO:0031023 | microtubule organizing center organization | 0.000848 | | P |
| GO:0005675 | holo TFIIH complex | 0.000859 | | C |
| GO:0005852 | eukaryotic translation initiation factor 3 complex | 0.00087 | | C |
| GO:0031401 | positive regulation of protein modification process | 0.000884 | | P |
| GO:0005847 | mRNA cleavage and polyadenylation specificity factor complex | 0.000902 | | C |
| GO:0008298 | intracellular mRNA localization | 0.000923 | | P |
| GO:0043410 | positive regulation of MAPK cascade | 0.000981 | | P |
| GO:1905952 | regulation of lipid localization | 0.000989 | | P |
| GO:0016197 | endosomal transport | 0.001 | | P |
| GO:0016049 | cell growth | 0.001 | | P |
| GO:0010975 | regulation of neuron projection development | 0.00102 | | P |
| GO:0045196 | establishment or maintenance of neuroblast polarity | 0.00102 | | P |
| GO:0009056 | catabolic process | 0.00105 | | P |
| GO:0030004 | cellular monovalent inorganic cation homeostasis | 0.00106 | | P |
| GO:0030641 | regulation of cellular pH | 0.00106 | | P |
| GO:0019220 | regulation of phosphate metabolic process | 0.00106 | | P |
| GO:0051174 | regulation of phosphorus metabolic process | 0.00106 | | P |
| GO:0007269 | neurotransmitter secretion | 0.00106 | | P |
| GO:0098916 | anterograde trans-synaptic signaling | 0.00106 | | P |
| GO:0007268 | chemical synaptic transmission | 0.00106 | | P |
| GO:0032956 | regulation of actin cytoskeleton organization | 0.00107 | | P |
| GO:0032970 | regulation of actin filament-based process | 0.00107 | | P |
| GO:0044708 | single-organism behavior | 0.00108 | | P |
| GO:0060491 | regulation of cell projection assembly | 0.00109 | | P |
| GO:0048589 | developmental growth | 0.00111 | | P |
| GO:0006403 | RNA localization | 0.00111 | | P |
| GO:0090303 | positive regulation of wound healing | 0.00113 | | P |
| GO:0048813 | dendrite morphogenesis | 0.00119 | | P |
| GO:0010628 | positive regulation of gene expression | 0.00123 | | P |
| GO:0006413 | translational initiation | 0.00128 | | P |
| GO:0032482 | Rab protein signal transduction | 0.0013 | | P |
| GO:0007009 | plasma membrane organization | 0.00138 | | P |
| GO:0009895 | negative regulation of catabolic process | 0.00139 | | P |
| GO:0051240 | positive regulation of multicellular organismal process | 0.00139 | | P |
| GO:0045202 | synapse | 0.00147 | | C |
| GO:0003712 | transcription cofactor activity | 0.00148 | | M |
| GO:0006457 | protein folding | 0.00149 | | P |
| GO:0010591 | regulation of lamellipodium assembly | 0.00151 | | P |
| GO:1902743 | regulation of lamellipodium organization | 0.00151 | | P |
| GO:1903532 | positive regulation of secretion by cell | 0.00153 | | P |
| GO:0097485 | neuron projection guidance | 0.00153 | | P |
| GO:0012505 | endomembrane system | 0.00157 | | C |
| GO:0035220 | wing disc development | 0.00164 | | P |
| GO:0010648 | negative regulation of cell communication | 0.00171 | | P |
| GO:0023057 | negative regulation of signaling | 0.00171 | | P |
| GO:0003779 | actin binding | 0.00172 | | M |
| GO:0051963 | regulation of synapse assembly | 0.00179 | | P |
| GO:0065009 | regulation of molecular function | 0.00189 | | P |
| GO:0060810 | intracellular mRNA localization involved in pattern specification process | 0.0019 | | P |
| GO:0051254 | positive regulation of RNA metabolic process | 0.00191 | | P |
| GO:0048639 | positive regulation of developmental growth | 0.00193 | | P |
| GO:0019899 | enzyme binding | 0.00195 | | M |
| GO:0060589 | nucleoside-triphosphatase regulator activity | 0.00196 | | M |
| GO:0015931 | nucleobase-containing compound transport | 0.00197 | | P |
| GO:0009628 | response to abiotic stimulus | 0.002 | | P |
| GO:0008285 | negative regulation of cell proliferation | 0.002 | | P |
| GO:0010970 | transport along microtubule | 0.00204 | | P |
| GO:0010506 | regulation of autophagy | 0.00204 | | P |
| GO:0099111 | microtubule-based transport | 0.00205 | | P |
| GO:0051173 | positive regulation of nitrogen compound metabolic process | 0.00209 | | P |
| GO:0004672 | protein kinase activity | 0.00215 | | M |
| GO:0061041 | regulation of wound healing | 0.00221 | | P |
| GO:0006379 | mRNA cleavage | 0.00223 | | P |
| GO:0005688 | U6 snRNP | 0.00225 | | C |
| GO:0035152 | regulation of tube architecture, open tracheal system | 0.00228 | | P |
| GO:0036099 | female germ-line stem cell population maintenance | 0.00232 | | P |
| GO:0006091 | generation of precursor metabolites and energy | 0.00234 | | P |
| GO:0007612 | learning | 0.00244 | | P |
| GO:0003002 | regionalization | 0.00248 | | P |
| GO:0006793 | phosphorus metabolic process | 0.00248 | | P |
| GO:0045664 | regulation of neuron differentiation | 0.00256 | | P |
| GO:0006796 | phosphate-containing compound metabolic process | 0.0026 | | P |
| GO:0016183 | synaptic vesicle coating | 0.00271 | | P |
| GO:0006901 | vesicle coating | 0.00272 | | P |
| GO:0005849 | mRNA cleavage factor complex | 0.00272 | | C |
| GO:0002682 | regulation of immune system process | 0.00275 | | P |
| GO:0035060 | brahma complex | 0.00281 | | C |
| GO:0046903 | secretion | 0.00292 | | P |
| GO:0001111 | promoter clearance from RNA polymerase II promoter | 0.00293 | | P |
| GO:0001109 | promoter clearance during DNA-templated transcription | 0.00294 | | P |
| GO:0097458 | neuron part | 0.00298 | | C |
| GO:0007423 | sensory organ development | 0.00305 | | P |
| GO:0010605 | negative regulation of macromolecule metabolic process | 0.00305 | | P |
| GO:0045935 | positive regulation of nucleobase-containing compound metabolic process | 0.00308 | | P |
| GO:0051248 | negative regulation of protein metabolic process | 0.00308 | | P |
| GO:0008150 | biological_process | 0.00308 | | P |
| GO:0010769 | regulation of cell morphogenesis involved in differentiation | 0.00309 | | P |
| GO:0032269 | negative regulation of cellular protein metabolic process | 0.00309 | | P |
| GO:0051169 | nuclear transport | 0.00315 | | P |
| GO:0006913 | nucleocytoplasmic transport | 0.00316 | | P |
| GO:0010557 | positive regulation of macromolecule biosynthetic process | 0.00329 | | P |
| GO:0045596 | negative regulation of cell differentiation | 0.00331 | | P |
| GO:0060627 | regulation of vesicle-mediated transport | 0.00338 | | P |
| GO:0080134 | regulation of response to stress | 0.00341 | | P |
| GO:0005700 | polytene chromosome | 0.00345 | | C |
| GO:0016241 | regulation of macroautophagy | 0.00349 | | P |
| GO:0050790 | regulation of catalytic activity | 0.00367 | | P |
| GO:0022416 | chaeta development | 0.00384 | | P |
| GO:0051493 | regulation of cytoskeleton organization | 0.00384 | | P |
| GO:0031532 | actin cytoskeleton reorganization | 0.00385 | | P |
| GO:0044437 | vacuolar part | 0.0042 | | C |
| GO:0000145 | exocyst | 0.0043 | | C |
| GO:0048598 | embryonic morphogenesis | 0.00435 | | P |
| GO:0072657 | protein localization to membrane | 0.00449 | | P |
| GO:0035295 | tube development | 0.0045 | | P |
| GO:0001745 | compound eye morphogenesis | 0.00452 | | P |
| GO:0008360 | regulation of cell shape | 0.00456 | | P |
| GO:0070646 | protein modification by small protein removal | 0.00457 | | P |
| GO:0032386 | regulation of intracellular transport | 0.00458 | | P |
| GO:0016482 | cytosolic transport | 0.00479 | | P |
| GO:0031328 | positive regulation of cellular biosynthetic process | 0.005 | | P |
| GO:0009891 | positive regulation of biosynthetic process | 0.00502 | | P |
| GO:0008306 | associative learning | 0.00512 | | P |
| GO:1903508 | positive regulation of nucleic acid-templated transcription | 0.00513 | | P |
| GO:0070372 | regulation of ERK1 and ERK2 cascade | 0.00514 | | P |
| GO:0045893 | positive regulation of transcription, DNA-templated | 0.00515 | | P |
| GO:1902680 | positive regulation of RNA biosynthetic process | 0.00516 | | P |
| GO:0030119 | AP-type membrane coat adaptor complex | 0.00529 | | C |
| GO:0032806 | carboxy-terminal domain protein kinase complex | 0.00533 | | C |
| GO:0090544 | BAF-type complex | 0.00539 | | C |
| GO:0044459 | plasma membrane part | 0.00542 | | C |
| GO:1904396 | regulation of neuromuscular junction development | 0.00543 | | P |
| GO:0050769 | positive regulation of neurogenesis | 0.00543 | | P |
| GO:0007444 | imaginal disc development | 0.00544 | | P |
| GO:0034334 | adherens junction maintenance | 0.00544 | | P |
| GO:0003743 | translation initiation factor activity | 0.00551 | | M |
| GO:0008090 | retrograde axonal transport | 0.00553 | | P |
| GO:0051235 | maintenance of location | 0.00556 | | P |
| GO:0005744 | mitochondrial inner membrane presequence translocase complex | 0.00558 | | C |
| GO:0051093 | negative regulation of developmental process | 0.00575 | | P |
| GO:0010507 | negative regulation of autophagy | 0.00577 | | P |
| GO:0097581 | lamellipodium organization | 0.00578 | | P |
| GO:0030032 | lamellipodium assembly | 0.00579 | | P |
| GO:0008187 | poly-pyrimidine tract binding | 0.00582 | | M |
| GO:0032446 | protein modification by small protein conjugation | 0.00603 | | P |
| GO:0007266 | Rho protein signal transduction | 0.00617 | | P |
| GO:0008180 | COP9 signalosome | 0.00617 | | C |
| GO:0032880 | regulation of protein localization | 0.0066 | | P |
| GO:0005768 | endosome | 0.00671 | | C |
| GO:1905369 | endopeptidase complex | 0.00672 | | C |
| GO:0000502 | proteasome complex | 0.0068 | | C |
| GO:0005057 | signal transducer activity, downstream of receptor | 0.00681 | | M |
| GO:0030695 | GTPase regulator activity | 0.00688 | | M |
| GO:0007349 | cellularization | 0.00691 | | P |
| GO:0051047 | positive regulation of secretion | 0.00692 | | P |
| GO:0006342 | chromatin silencing | 0.00698 | | P |
| GO:0007411 | axon guidance | 0.00716 | | P |
| GO:0000165 | MAPK cascade | 0.00748 | | P |
| GO:0023014 | signal transduction by protein phosphorylation | 0.00749 | | P |
| GO:0007406 | negative regulation of neuroblast proliferation | 0.00764 | | P |
| GO:0030589 | pseudocleavage involved in syncytial blastoderm formation | 0.00765 | | P |
| GO:0002793 | positive regulation of peptide secretion | 0.00766 | | P |
| GO:2000178 | negative regulation of neural precursor cell proliferation | 0.00766 | | P |
| GO:0030588 | pseudocleavage | 0.00767 | | P |
| GO:1903364 | positive regulation of cellular protein catabolic process | 0.00767 | | P |
| GO:0009416 | response to light stimulus | 0.00783 | | P |
| GO:0022892 | substrate-specific transporter activity | 0.00794 | | M |
| GO:0007274 | neuromuscular synaptic transmission | 0.008 | | P |
| GO:0008536 | Ran GTPase binding | 0.00808 | | M |
| GO:1901981 | phosphatidylinositol phosphate binding | 0.00809 | | M |
| GO:1905368 | peptidase complex | 0.00816 | | C |
| GO:0033365 | protein localization to organelle | 0.00872 | | P |
| GO:0009896 | positive regulation of catabolic process | 0.00908 | | P |
| GO:0032785 | negative regulation of DNA-templated transcription, elongation | 0.0091 | | P |
| GO:0051222 | positive regulation of protein transport | 0.00911 | | P |
| GO:0099513 | polymeric cytoskeletal fiber | 0.00917 | | C |
| GO:0008541 | proteasome regulatory particle, lid subcomplex | 0.00919 | | C |
| GO:0045944 | positive regulation of transcription from RNA polymerase II promoter | 0.00923 | | P |
| GO:0000226 | microtubule cytoskeleton organization | 0.00928 | | P |
| GO:0051490 | negative regulation of filopodium assembly | 0.00929 | | P |
| GO:0016772 | transferase activity, transferring phosphorus-containing groups | 0.00934 | | M |
| GO:0009605 | response to external stimulus | 0.00936 | | P |
| GO:0070374 | positive regulation of ERK1 and ERK2 cascade | 0.00946 | | P |
| GO:0005884 | actin filament | 0.00949 | | C |
| GO:0098529 | neuromuscular junction development, skeletal muscle fiber | 0.00969 | | P |
| GO:0031331 | positive regulation of cellular catabolic process | 0.00979 | | P |
| GO:0045747 | positive regulation of Notch signaling pathway | 0.00986 | | P |
| GO:0045814 | negative regulation of gene expression, epigenetic | 0.0099 | | P |
| GO:0030120 | vesicle coat | 0.00994 | | C |
| GO:1901701 | cellular response to oxygen-containing compound | 0.00997 | | P |
| GO:0045666 | positive regulation of neuron differentiation | 0.01 | | P |
| GO:0099512 | supramolecular fiber | 0.01 | | C |
| GO:0061695 | transferase complex, transferring phosphorus-containing groups | 0.01 | | C |
| GO:0030117 | membrane coat | 0.0101 | | C |
| GO:0099081 | supramolecular polymer | 0.0101 | | C |
| GO:0007283 | spermatogenesis | 0.0102 | | P |
| GO:0009314 | response to radiation | 0.0102 | | P |
| GO:0048193 | Golgi vesicle transport | 0.0102 | | P |
| GO:0051241 | negative regulation of multicellular organismal process | 0.0102 | | P |
| GO:0005956 | protein kinase CK2 complex | 0.0102 | | C |
| GO:0022890 | inorganic cation transmembrane transporter activity | 0.0103 | | M |
| GO:0001112 | DNA-templated transcriptional open complex formation | 0.0104 | | P |
| GO:0035148 | tube formation | 0.0105 | | P |
| GO:0008586 | imaginal disc-derived wing vein morphogenesis | 0.0105 | | P |
| GO:0034367 | macromolecular complex remodeling | 0.0105 | | P |
| GO:0001113 | transcriptional open complex formation at RNA polymerase II promoter | 0.0105 | | P |
| GO:0001120 | protein-DNA complex remodeling | 0.0105 | | P |
| GO:0060341 | regulation of cellular localization | 0.0105 | | P |
| GO:0042176 | regulation of protein catabolic process | 0.0108 | | P |
| GO:0016055 | Wnt signaling pathway | 0.0108 | | P |
| GO:1905114 | cell surface receptor signaling pathway involved in cell-cell signaling | 0.0108 | | P |
| GO:0016319 | mushroom body development | 0.0109 | | P |
| GO:1901575 | organic substance catabolic process | 0.0109 | | P |
| GO:0090087 | regulation of peptide transport | 0.011 | | P |
| GO:0042623 | ATPase activity, coupled | 0.0111 | | M |
| GO:0010906 | regulation of glucose metabolic process | 0.0117 | | P |
| GO:0009889 | regulation of biosynthetic process | 0.0117 | | P |
| GO:0003674 | molecular_function | 0.0117 | | M |
| GO:0044089 | positive regulation of cellular component biogenesis | 0.0118 | | P |
| GO:0019932 | second-messenger-mediated signaling | 0.012 | | P |
| GO:0048232 | male gamete generation | 0.012 | | P |
| GO:0016180 | snRNA processing | 0.0121 | | P |
| GO:1904951 | positive regulation of establishment of protein localization | 0.0126 | | P |
| GO:2000112 | regulation of cellular macromolecule biosynthetic process | 0.0126 | | P |
| GO:0018193 | peptidyl-amino acid modification | 0.0127 | | P |
| GO:0043254 | regulation of protein complex assembly | 0.0127 | | P |
| GO:0005048 | signal sequence binding | 0.0154 | | M |
| GO:0008092 | cytoskeletal protein binding | 0.0154 | | M |
| GO:0000146 | microfilament motor activity | 0.0165 | | M |
| GO:0070594 | juvenile hormone response element binding | 0.0165 | | M |
| GO:0017016 | Ras GTPase binding | 0.0168 | | M |
| GO:0051020 | GTPase binding | 0.0203 | | M |
| GO:0044877 | macromolecular complex binding | 0.0204 | | M |
| GO:0098772 | molecular function regulator | 0.0214 | | M |
| GO:0008324 | cation transmembrane transporter activity | 0.0216 | | M |
| GO:0016773 | phosphotransferase activity, alcohol group as acceptor | 0.0218 | | M |
| GO:0031267 | small GTPase binding | 0.0224 | | M |
| GO:0019900 | kinase binding | 0.0233 | | M |
| GO:0030234 | enzyme regulator activity | 0.0243 | | M |
| GO:0005243 | gap junction channel activity | 0.0244 | | M |
| GO:0016301 | kinase activity | 0.0246 | | M |
| GO:0022829 | wide pore channel activity | 0.0247 | | M |
| GO:0005096 | GTPase activator activity | 0.0254 | | M |
| GO:0099094 | ligand-gated cation channel activity | 0.0256 | | M |
| GO:0004842 | ubiquitin-protein transferase activity | 0.0257 | | M |
| GO:0004702 | signal transducer, downstream of receptor, with serine/threonine kinase activity | 0.0266 | | M |

**Supplemental Table 11 |** Significantly differentiated genes shared between all population comparisons (DM gene name = *D. montana* gene name, FBN_vir = FlyBase gene number for *D. virilis* ortholog, FBGN_vir = FlyBase gene name for *D. virilis* ortholog, FBN_mel = FlyBase gene number for *D. melanogaster* ortholog, FBGN_mel = FlyBase gene name for *D. melanogaster* ortholog)

| **DM gene name** | **FBN_vir** | **FBGN_vir** | **FBN_mel** | **FBGN_mel** |
| --- | --- | --- | --- | --- |
| **snap_masked-scaffold235-size190281-abinit-gene-0.10** | NA | NA | NA | NA |
| **maker-scaffold852-size125918-augustus-gene-1.5** | FBgn0203117 | Dvir\GJ15930 | FBgn0028516 | ZnT35C |
| **snap_masked-scaffold1202-size36259-abinit-gene-0.4** | FBgn0205554 | Dvir\GJ18395 | FBgn0000043 | Act42A |
| **snap_masked-scaffold2536-size60091-abinit-gene-0.4** | FBgn0204001 | Dvir\GJ16822 | FBgn0264598 | PsGEF |
| **maker-scaffold657-size51373-snap-gene-0.10** | FBgn0198868 | Dvir\GJ11611 | FBgn0086690 | Plp |
| **augustus_masked-scaffold235-size190281-abinit-gene-0.5** | NA | NA | NA | NA |
| **maker-scaffold2845-size21498-snap-gene-0.8** | FBgn0203984 | Dvir\GJ16805 | FBgn0030588;  FBgn0030589 | CG9521;  CG9519 |
| **augustus_masked-scaffold606-size110555-abinit-gene-1.1** | FBgn0201457 | Dvir\GJ14242 | FBgn0051619 | nolo |
| **maker-scaffold231-size39745-snap-gene-0.21** | FBgn0205458 | Dvir\GJ18297 | FBgn0032180 | CG44153 |
| **maker-scaffold26-size169467-snap-gene-0.26** | FBgn0206766 | Dvir\GJ19624 | FBgn0031292 | CG15824 |

**Supplemental Table 12 | Significantly (FDR < 0.1) enriched GO-terms for Colorado: Oulanka** population comparisons

| **GO term** | **GO term description** | **FDR** | **GO type** |
| --- | --- | --- | --- |
| **GO:0044459** | plasma membrane part | 0.00000291 | C |
| **GO:0031224** | intrinsic component of membrane | 0.000037 | C |
| **GO:0016021** | integral component of membrane | 0.0000411 | C |
| **GO:0007610** | behavior | 0.00048 | P |
| **GO:0044425** | membrane part | 0.000698 | C |
| **GO:0031226** | intrinsic component of plasma membrane | 0.000786 | C |
| **GO:0005886** | plasma membrane | 0.00128 | C |
| **GO:0030054** | cell junction | 0.00133 | C |
| **GO:0005887** | integral component of plasma membrane | 0.00144 | C |
| **GO:0005924** | cell-substrate adherens junction | 0.00147 | C |
| **GO:0030055** | cell-substrate junction | 0.00166 | C |
| **GO:0005524** | ATP binding | 0.00467 | M |
| **GO:0030554** | adenyl nucleotide binding | 0.00481 | M |
| **GO:0043167** | ion binding | 0.00494 | M |
| **GO:0005912** | adherens junction | 0.0055 | C |
| **GO:0043168** | anion binding | 0.00571 | M |
| **GO:0070161** | anchoring junction | 0.006 | C |
| **GO:0032559** | adenyl ribonucleotide binding | 0.00654 | M |
| **GO:0004872** | receptor activity | 0.0088 | M |
| **GO:0060089** | molecular transducer activity | 0.00957 | M |
| **GO:0097367** | carbohydrate derivative binding | 0.00972 | M |
| **GO:0008144** | drug binding | 0.00997 | M |
| **GO:0051959** | dynein light intermediate chain binding | 0.0101 | M |
| **GO:0015318** | inorganic molecular entity transmembrane transporter activity | 0.0103 | M |
| **GO:0016887** | ATPase activity | 0.0124 | M |
| **GO:0042623** | ATPase activity, coupled | 0.0127 | M |
| **GO:0032553** | ribonucleotide binding | 0.0129 | M |
| **GO:0016020** | membrane | 0.0146 | C |
| **GO:0000166** | nucleotide binding | 0.0152 | M |
| **GO:1901265** | nucleoside phosphate binding | 0.0162 | M |
| **GO:0032555** | purine ribonucleotide binding | 0.019 | M |
| **GO:0017076** | purine nucleotide binding | 0.0209 | M |
| **GO:0004871** | signal transducer activity | 0.0211 | M |
| **GO:0035639** | purine ribonucleoside triphosphate binding | 0.0218 | M |
| **GO:0003008** | system process | 0.0222 | P |
| **GO:0050808** | synapse organization | 0.0231 | P |
| **GO:0007611** | learning or memory | 0.0237 | P |
| **GO:0022857** | transmembrane transporter activity | 0.0242 | M |
| **GO:0043492** | ATPase activity, coupled to movement of substances | 0.0252 | M |
| **GO:0051128** | regulation of cellular component organization | 0.0253 | P |
| **GO:0120025** | plasma membrane bounded cell projection | 0.0258 | C |
| **GO:0050890** | cognition | 0.0271 | P |
| **GO:0042995** | cell projection | 0.0284 | C |
| **GO:0008074** | guanylate cyclase complex, soluble | 0.0298 | C |
| **GO:0050794** | regulation of cellular process | 0.031 | P |
| **GO:0035556** | intracellular signal transduction | 0.0317 | P |
| **GO:0045503** | dynein light chain binding | 0.0333 | M |
| **GO:0044456** | synapse part | 0.0341 | C |
| **GO:0065007** | biological regulation | 0.0357 | P |
| **GO:0046068** | cGMP metabolic process | 0.0369 | P |
| **GO:2000026** | regulation of multicellular organismal development | 0.0454 | P |
| **GO:0004034** | aldose 1-epimerase activity | 0.0464 | M |
| **GO:0036094** | small molecule binding | 0.0468 | M |
| **GO:0004706** | JUN kinase kinase kinase activity | 0.047 | M |
| **GO:0061689** | tricellular tight junction | 0.0539 | C |
| **GO:0009187** | cyclic nucleotide metabolic process | 0.0554 | P |
| **GO:0044087** | regulation of cellular component biogenesis | 0.0555 | P |
| **GO:0007165** | signal transduction | 0.0585 | P |
| **GO:0050877** | nervous system process | 0.0626 | P |
| **GO:0015294** | solute:cation symporter activity | 0.0641 | M |
| **GO:0004383** | guanylate cyclase activity | 0.065 | M |
| **GO:0050793** | regulation of developmental process | 0.0669 | P |
| **GO:0007613** | memory | 0.0684 | P |
| **GO:1990709** | presynaptic active zone organization | 0.0693 | P |
| **GO:0050789** | regulation of biological process | 0.0718 | P |
| **GO:0022838** | substrate-specific channel activity | 0.0768 | M |
| **GO:0010499** | proteasomal ubiquitin-independent protein catabolic process | 0.0814 | P |
| **GO:0065008** | regulation of biological quality | 0.0896 | P |
| **GO:1904071** | presynaptic active zone assembly | 0.0904 | P |
| **GO:0006182** | cGMP biosynthetic process | 0.0912 | P |
| **GO:0006171** | cAMP biosynthetic process | 0.0914 | P |
| **GO:2000809** | positive regulation of synaptic vesicle clustering | 0.0939 | P |
| **GO:0023051** | regulation of signaling | 0.0965 | P |
| **GO:2000807** | regulation of synaptic vesicle clustering | 0.0976 | P |

**Supplemental Table 13 | Significantly (FDR < 0.1) enriched GO-terms for Colorado: Vancouver** population comparisons

| **GO term** | **GO term description** | **FDR** | **GO type** |
| --- | --- | --- | --- |
| **GO:0005887** | integral component of plasma membrane | 0.0000187 | C |
| **GO:0031226** | intrinsic component of plasma membrane | 0.000026 | C |
| **GO:0031224** | intrinsic component of membrane | 0.0000303 | C |
| **GO:0120025** | plasma membrane bounded cell projection | 0.0000348 | C |
| **GO:0004872** | receptor activity | 0.0000354 | M |
| **GO:0016021** | integral component of membrane | 0.000036 | C |
| **GO:0042995** | cell projection | 0.0000439 | C |
| **GO:0044459** | plasma membrane part | 0.0000448 | C |
| **GO:0060089** | molecular transducer activity | 0.0000575 | M |
| **GO:0097458** | neuron part | 0.000441 | C |
| **GO:0038023** | signaling receptor activity | 0.000694 | M |
| **GO:0004871** | signal transducer activity | 0.00154 | M |
| **GO:0097485** | neuron projection guidance | 0.00162 | P |
| **GO:0043005** | neuron projection | 0.0018 | C |
| **GO:0044425** | membrane part | 0.00258 | C |
| **GO:0004888** | transmembrane signaling receptor activity | 0.0029 | M |
| **GO:0006928** | movement of cell or subcellular component | 0.00291 | P |
| **GO:0007411** | axon guidance | 0.00297 | P |
| **GO:0099600** | transmembrane receptor activity | 0.00348 | M |
| **GO:0030424** | axon | 0.00487 | C |
| **GO:0005524** | ATP binding | 0.00488 | M |
| **GO:0032559** | adenyl ribonucleotide binding | 0.00495 | M |
| **GO:0046873** | metal ion transmembrane transporter activity | 0.00524 | M |
| **GO:0030554** | adenyl nucleotide binding | 0.0053 | M |
| **GO:0008144** | drug binding | 0.00559 | M |
| **GO:0000166** | nucleotide binding | 0.013 | M |
| **GO:1901265** | nucleoside phosphate binding | 0.0141 | M |
| **GO:0044463** | cell projection part | 0.0144 | C |
| **GO:0120038** | plasma membrane bounded cell projection part | 0.0156 | C |
| **GO:1902495** | transmembrane transporter complex | 0.017 | C |
| **GO:0015347** | sodium-independent organic anion transmembrane transporter activity | 0.0205 | M |
| **GO:0005102** | receptor binding | 0.0236 | M |
| **GO:0036094** | small molecule binding | 0.0238 | M |
| **GO:0030155** | regulation of cell adhesion | 0.0272 | P |
| **GO:0097367** | carbohydrate derivative binding | 0.0297 | M |
| **GO:0061176** | type Ib terminal bouton | 0.0377 | C |
| **GO:0032553** | ribonucleotide binding | 0.0436 | M |
| **GO:1905862** | ferroxidase complex | 0.0436 | C |
| **GO:0016328** | lateral plasma membrane | 0.0453 | C |
| **GO:0033573** | high-affinity iron permease complex | 0.0463 | C |
| **GO:0008092** | cytoskeletal protein binding | 0.0488 | M |
| **GO:0043168** | anion binding | 0.0497 | M |
| **GO:0048608** | reproductive structure development | 0.0553 | P |
| **GO:0016020** | membrane | 0.0561 | C |
| **GO:0008037** | cell recognition | 0.0604 | P |
| **GO:0050818** | regulation of coagulation | 0.0626 | P |
| **GO:0035639** | purine ribonucleoside triphosphate binding | 0.0637 | M |
| **GO:0015079** | potassium ion transmembrane transporter activity | 0.0644 | M |
| **GO:0050819** | negative regulation of coagulation | 0.0678 | P |
| **GO:0017076** | purine nucleotide binding | 0.0704 | M |
| **GO:0032555** | purine ribonucleotide binding | 0.0729 | M |
| **GO:0061045** | negative regulation of wound healing | 0.074 | P |
| **GO:2001023** | regulation of response to drug | 0.0803 | P |
| **GO:0030195** | negative regulation of blood coagulation | 0.0814 | P |
| **GO:0016301** | kinase activity | 0.0829 | M |
| **GO:0007166** | cell surface receptor signaling pathway | 0.0863 | P |
| **GO:0008038** | neuron recognition | 0.087 | P |
| **GO:0007165** | signal transduction | 0.0891 | P |
| **GO:0008406** | gonad development | 0.0895 | P |
| **GO:0004713** | protein tyrosine kinase activity | 0.09 | M |
| **GO:0030193** | regulation of blood coagulation | 0.0904 | P |
| **GO:0008045** | motor neuron axon guidance | 0.0915 | P |
| **GO:0005088** | Ras guanyl-nucleotide exchange factor activity | 0.0929 | M |
| **GO:0006189** | 'de novo' IMP biosynthetic process | 0.0937 | P |
| **GO:0007178** | transmembrane receptor protein serine/threonine kinase signaling pathway | 0.0938 | P |
| **GO:0005261** | cation channel activity | 0.0948 | M |

**Supplemental Table 14 | Significantly (FDR < 0.1) enriched GO-terms for Oulanka: Vancouver** population comparisons

| **GO term** | **GO term description** | **FDR** | **GO type** |
| --- | --- | --- | --- |
| **GO:0005886** | plasma membrane | 0.0000765 | C |
| **GO:0016020** | membrane | 0.000127 | C |
| **GO:0044459** | plasma membrane part | 0.000175 | C |
| **GO:0031224** | intrinsic component of membrane | 0.000548 | C |
| **GO:0016021** | integral component of membrane | 0.000637 | C |
| **GO:0065007** | biological regulation | 0.00225 | P |
| **GO:0060089** | molecular transducer activity | 0.00379 | M |
| **GO:0004871** | signal transducer activity | 0.0041 | M |
| **GO:0050793** | regulation of developmental process | 0.00426 | P |
| **GO:0050789** | regulation of biological process | 0.00442 | P |
| **GO:0050794** | regulation of cellular process | 0.00448 | P |
| **GO:0043167** | ion binding | 0.00485 | M |
| **GO:0098590** | plasma membrane region | 0.00502 | C |
| **GO:2000026** | regulation of multicellular organismal development | 0.00521 | P |
| **GO:0051128** | regulation of cellular component organization | 0.0055 | P |
| **GO:0004872** | receptor activity | 0.00577 | M |
| **GO:0040008** | regulation of growth | 0.00653 | P |
| **GO:0050808** | synapse organization | 0.0066 | P |
| **GO:0036156** | inner dynein arm | 0.00713 | C |
| **GO:0044425** | membrane part | 0.00734 | C |
| **GO:0051239** | regulation of multicellular organismal process | 0.00922 | P |
| **GO:0051960** | regulation of nervous system development | 0.0124 | P |
| **GO:0038023** | signaling receptor activity | 0.013 | M |
| **GO:0030554** | adenyl nucleotide binding | 0.0133 | M |
| **GO:0140135** | mechanosensitive cation channel activity | 0.0133 | M |
| **GO:0001539** | cilium or flagellum-dependent cell motility | 0.0136 | P |
| **GO:0032559** | adenyl ribonucleotide binding | 0.014 | M |
| **GO:0004888** | transmembrane signaling receptor activity | 0.0143 | M |
| **GO:0005524** | ATP binding | 0.0146 | M |
| **GO:0042623** | ATPase activity, coupled | 0.0146 | M |
| **GO:0099600** | transmembrane receptor activity | 0.0153 | M |
| **GO:0008047** | enzyme activator activity | 0.0155 | M |
| **GO:0046872** | metal ion binding | 0.016 | M |
| **GO:0016887** | ATPase activity | 0.0161 | M |
| **GO:0035639** | purine ribonucleoside triphosphate binding | 0.0162 | M |
| **GO:0022857** | transmembrane transporter activity | 0.0167 | M |
| **GO:0005215** | transporter activity | 0.0169 | M |
| **GO:0032555** | purine ribonucleotide binding | 0.0174 | M |
| **GO:0015318** | inorganic molecular entity transmembrane transporter activity | 0.0186 | M |
| **GO:0043547** | positive regulation of GTPase activity | 0.0187 | P |
| **GO:0015075** | ion transmembrane transporter activity | 0.0191 | M |
| **GO:0017076** | purine nucleotide binding | 0.0195 | M |
| **GO:0000166** | nucleotide binding | 0.0195 | M |
| **GO:0043169** | cation binding | 0.0198 | M |
| **GO:1901265** | nucleoside phosphate binding | 0.0203 | M |
| **GO:0008144** | drug binding | 0.0206 | M |
| **GO:0005319** | lipid transporter activity | 0.0206 | M |
| **GO:0032553** | ribonucleotide binding | 0.0207 | M |
| **GO:0097367** | carbohydrate derivative binding | 0.0208 | M |
| **GO:0004649** | poly(ADP-ribose) glycohydrolase activity | 0.0209 | M |
| **GO:0048869** | cellular developmental process | 0.0229 | P |
| **GO:0008324** | cation transmembrane transporter activity | 0.0231 | M |
| **GO:0043168** | anion binding | 0.0251 | M |
| **GO:0051959** | dynein light intermediate chain binding | 0.0252 | M |
| **GO:0046914** | transition metal ion binding | 0.0285 | M |
| **GO:0000923** | equatorial microtubule organizing center | 0.0291 | C |
| **GO:0036094** | small molecule binding | 0.0293 | M |
| **GO:0034702** | ion channel complex | 0.0331 | C |
| **GO:0051961** | negative regulation of nervous system development | 0.0344 | P |
| **GO:0030054** | cell junction | 0.0367 | C |
| **GO:0050807** | regulation of synapse organization | 0.0406 | P |
| **GO:0008275** | gamma-tubulin small complex | 0.0407 | C |
| **GO:0035556** | intracellular signal transduction | 0.0411 | P |
| **GO:0051963** | regulation of synapse assembly | 0.0413 | P |
| **GO:0048638** | regulation of developmental growth | 0.0417 | P |
| **GO:0043087** | regulation of GTPase activity | 0.0423 | P |
| **GO:0008331** | high voltage-gated calcium channel activity | 0.0436 | M |
| **GO:0032502** | developmental process | 0.0447 | P |
| **GO:0045503** | dynein light chain binding | 0.0486 | M |
| **GO:0045595** | regulation of cell differentiation | 0.0501 | P |
| **GO:0051011** | microtubule minus-end binding | 0.0582 | M |
| **GO:0043015** | gamma-tubulin binding | 0.0597 | M |
| **GO:0046873** | metal ion transmembrane transporter activity | 0.0607 | M |
| **GO:0060284** | regulation of cell development | 0.0676 | P |
| **GO:0051415** | interphase microtubule nucleation by interphase microtubule organizing center | 0.0703 | P |
| **GO:0031523** | Myb complex | 0.0719 | C |
| **GO:0097060** | synaptic membrane | 0.073 | C |
| **GO:0048583** | regulation of response to stimulus | 0.0769 | P |
| **GO:0065008** | regulation of biological quality | 0.0802 | P |
| **GO:1904396** | regulation of neuromuscular junction development | 0.092 | P |
| **GO:0009653** | anatomical structure morphogenesis | 0.0944 | P |
| **GO:0048477** | oogenesis | 0.0944 | P |
| **GO:0060285** | cilium-dependent cell motility | 0.095 | P |
| **GO:0003008** | system process | 0.0953 | P |
| **GO:0007165** | signal transduction | 0.0964 | P |
| **GO:0050803** | regulation of synapse structure or activity | 0.0971 | P |
| **GO:0008039** | synaptic target recognition | 0.0975 | P |
| **GO:0007200** | phospholipase C-activating G-protein coupled receptor signaling pathway | 0.0978 | P |
| **GO:0009306** | protein secretion | 0.0979 | P |
| **GO:0033044** | regulation of chromosome organization | 0.0984 | P |
| **GO:0022603** | regulation of anatomical structure morphogenesis | 0.0992 | P |

**Supplemental Table 15 | Significantly enriched GO-terms shared between all population comparisons**

| GO term | GO term description | FDR (Colorado: Oulanka) | FDR (Colorado: Vancouver) | FDR (Oulanka: Vancouver) |
| --- | --- | --- | --- | --- |
| **GO:0044459** | plasma membrane part | 0.00000291 | 0.0000448 | 0.000175 |
| **GO:0031224** | intrinsic component of membrane | 0.000037 | 0.0000303 | 0.000548 |
| **GO:0016021** | integral component of membrane | 0.0000411 | 0.000036 | 0.000637 |
| **GO:0044425** | membrane part | 0.000698 | 0.00258 | 0.00734 |
| **GO:0060089** | molecular transducer activity | 0.00957 | 0.0000575 | 0.00379 |
| **GO:0004872** | receptor activity | 0.0088 | 0.0000354 | 0.00577 |
| **GO:0030554** | adenyl nucleotide binding | 0.00481 | 0.0053 | 0.0133 |
| **GO:0005524** | ATP binding | 0.00467 | 0.00488 | 0.0146 |
| **GO:0032559** | adenyl ribonucleotide binding | 0.00654 | 0.00495 | 0.014 |
| **GO:0004871** | signal transducer activity | 0.0211 | 0.00154 | 0.0041 |
| **GO:0008144** | drug binding | 0.00997 | 0.00559 | 0.0206 |
| **GO:0000166** | nucleotide binding | 0.0152 | 0.013 | 0.0195 |
| **GO:1901265** | nucleoside phosphate binding | 0.0162 | 0.0141 | 0.0203 |
| **GO:0097367** | carbohydrate derivative binding | 0.00972 | 0.0297 | 0.0208 |
| **GO:0016020** | membrane | 0.0146 | 0.0561 | 0.000127 |
| **GO:0032553** | ribonucleotide binding | 0.0129 | 0.0436 | 0.0207 |
| **GO:0043168** | anion binding | 0.00571 | 0.0497 | 0.0251 |
| **GO:0036094** | small molecule binding | 0.0468 | 0.0238 | 0.0293 |
| **GO:0035639** | purine ribonucleoside triphosphate binding | 0.0218 | 0.0637 | 0.0162 |
| **GO:0032555** | purine ribonucleotide binding | 0.019 | 0.0729 | 0.0174 |
| **GO:0017076** | purine nucleotide binding | 0.0209 | 0.0704 | 0.0195 |
| **GO:0007165** | signal transduction | 0.0585 | 0.0891 | 0.0964 |

**Supplemental Table 16 | Significantly (FDR < 0.1) enriched functional clusters for Colorado: Oulanka** population comparisons

| **Cluster** | **Enrichment** | **P** | **Annotation terms** |
| --- | --- | --- | --- |
| **1** | 4.45 | 3.55E-05 | Nucleotide-binding, ATP-binding |
| **2** | 3.20 | 0.0006 | Membrane, Transmembrane helix, Integral component of membrane, Transmembrane |
| **3** | 2.09 | 0.0082 | ABC transporter, coupled to transmembrane movement of substances, ATPase activity, transmembrane domain |
| **4** | 2.00 | 0.0100 | GcAMP biosynthetic process, Adenylyl cyclase class-3/4/guanylyl cyclase, adenylate cyclase activity, cAMP-mediated signaling |
| **5** | 1.93 | 0.0117 | Low-density lipoprotein (LDL) receptor class A repeat, Low-density lipoprotein (LDL) receptor class A |
| **6** | 1.67 | 0.0213 | Zinc finger, Six-bladed beta-propeller, NHL repeat, TolB-like |
| **7** | 1.62 | 0.0239 | lipid transport, coupled to transmembrane movement of substances, ATPase activity, ABC transporter A |
| **8** | 1.52 | 0.0302 | Immunoglobulin domain, Immunoglobulin-like domain, Immunoglobulin I-set, Fibronectin, type III, Immunoglobulin-like fold |
| **9** | 1.41 | 0.0387 | protein serine/threonine kinase activity, ATP binding site, protein phosphorylation, nucleotide phosphate-binding region:ATP |
| **10** | 1.40 | 0.0399 | regulation of GTPase activity, GTPase activator activity, Rho GTPase activation protein |
| **11** | 1.37 | 0.0422 | chloride transport, chloride channel activity, chloride channel complex |
| **12** | 1.21 | 0.0612 | Receptor, Cell membrane |
| **13** | 1.21 | 0.0622 | guanylate cyclase complex, cGMP biosynthesis, Lyase, Purine metabolism, cGMP-mediated signaling, guanylate cyclase activity |
| **14** | 1.19 | 0.0644 | Flavoprotein, ecdysteroid metabolic process, FAD/NAD(P)-binding domain, Glucose-methanol-choline oxidoreductase, acting on CH-OH group of donors, Pyridine nucleotide-disulphide oxidoreductase, flavin adenine dinucleotide binding, oxidoreductase activity, choline dehydrogenase activity |
| **15** | 1.11 | 0.0769 | Glycoprotein, transmembrane region, glycosylation site:N-linked (GlcNAc...) |
| **16** | 1.11 | 0.0775 | ATPase activity, coiled coil stalk, cilium movement, ATP-dependent microtubule motor activity, ATPase, Dynein heavy chain domain, Cytoskeleton, microtubule-based movement |
| **17** | 1.05 | 0.0882 | Wnt signaling pathway |
| **18** | 1.05 | 0.0896 | transporter activity, ABC transporter A, transport |
| **19** | 1.04 | 0.0909 | Oxidoreductase FAD/NAD(P)-binding, Riboflavin synthase-like beta-barrel, Flavodoxin/nitric oxide synthase, Flavodoxin, Ferredoxin reductase-type FAD-binding domain, NADPH-cytochrome p450 reductase, FMN binding, NADP binding, FAD-binding, Flavoprotein pyridine nucleotide cytochrome reductase |
| **20** | 1.03 | 0.0932 | actomyosin contractile ring contraction, meiosis II cytokinesis, meiosis I cytokinesis, meiotic spindle organization |
| **21** | 1.03 | 0.0939 | nucleic acid binding, Zinc finger, Zinc finger C2H2-type/integrase DNA-binding domain |

**Supplemental Table 17 | Significantly (FDR < 0.1) enriched functional clusters for Colorado: Vancouver** population comparisons

| **Cluster** | **Enrichment** | **P** | **Annotation terms** |
| --- | --- | --- | --- |
| **1** | 1.98 | 0.0106 | integral component of membrane, Transmembrane, Membrane, Transmembrane helix |
| **2** | 1.90 | 0.0125 | Ras guanine nucleotide exchange factor, Guanine-nucleotide dissociation stimulator,small GTPase mediated signal transduction |
| **3** | 1.42 | 0.0380 | Alternative splicing, Phosphoprotein |
| **4** | 1.19 | 0.0650 | ATP-binding, Kinase, Nucleotide-binding, nucleotide phosphate-binding region:ATP |
| **5** | 1.06 | 0.0875 | Immunoglobulin I-set,synapse organization, Immunoglobulin subtype, Immunoglobulin subtype 2, Immunoglobulin-like domain |
| **6** | 1.00 | 0.0100 | protein ubiquitination, SCF ubiquitin ligase complex, Ubl conjugation pathway, Ubiquitin mediated proteolysis, Protein processing in endoplasmic reticulum |

**Supplemental Table 18 | Significantly (FDR < 0.1) enriched functional clusters for Oulanka: Vancouver** population comparisons

| **Cluster** | **Enrichment** | **P** | **Annotation terms** |
| --- | --- | --- | --- |
| **1** | 3.98 | 0.0001 | Nucleotide-binding, ATP-binding |
| **2** | 3.02 | 0.0010 | Membrane, Transmembrane helix, Integral component of membrane, Transmembrane |
| **3** | 2.05 | 0.0090 | ABC transporter-like, AAA+ ATPase domain |
| **4** | 1.91 | 0.0122 | Dynein heavy chain, ATPase activity, microtubule-based movement, Cytoskeleton, ATP-dependent microtubule motor activity |
| **5** | 1.56 | 0.0278 | Immunoglobulin-like domain, Cell adhesion, immunoglobulin C2-set, Fibronectin |
| **6** | 1.42 | 0.0381 | Epidermal growth factor-like domain, Insulin-like growth factor binding protein, Low-density lipoprotein (LDL) receptor class A, TolB-like |
| **7** | 1.35 | 0.0451 | coupled to transmembrane movement of substances, ABC transporter A, ATPase activity, transporter activity, lipid transport |
| **8** | 1.32 | 0.0483 | Postsynaptic cell membrane, Neurotransmitter-gated ion-channel ligand-binding, Cell junction, Gamma-aminobutyric acid A receptor, Neurotransmitter-gated ion-channel |
| **9** | 1.11 | 0.0785 | RhoGEF, Rho guanyl-nucleotide exchange factor activity, regulation of Rho protein signal transduction, Dbl homology (DH) domain |
| **10** | 1.05 | 0.0886 | RNA interference, Argonaute/Dicer protein |
| **11** | 1.04 | 0.0919 | sterol-transporting ATPase activity, Defense mechanisms, ABC-2 type transporter, cholesterol transporter activity, ABC transporter |
| **12** | 1.02 | 0.0952 | RNA export from nucleus, nuclear localization sequence binding, nucleocytoplasmic transporter activity, SMAD protein import into nucleus |
| **13** | 1.01 | 0.0980 | Cytoskeleton, spindle pole, microtubule, microtubule-based process, Microtubule binding |
